# Hybrid Multivalent Jack Bean α-Mannosidase Inhibitors: The First Example of Gold Nanoparticles Decorated with Deoxynojirimycin Inhitopes

**DOI:** 10.3390/molecules26195864

**Published:** 2021-09-27

**Authors:** Costanza Vanni, Anne Bodlenner, Marco Marradi, Jérémy P. Schneider, Maria de los Angeles Ramirez, Sergio Moya, Andrea Goti, Francesca Cardona, Philippe Compain, Camilla Matassini

**Affiliations:** 1 Dipartimento di Chimica “Ugo Schiff”, Università di Firenze, Via della Lastruccia 3-13, 50019 Sesto Fiorentino, Italy; costanza.vanni@unifi.it (C.V.); marco.marradi@unifi.it (M.M.); andrea.goti@unifi.it (A.G.); francesca.cardona@unifi.it (F.C.); 2 Laboratoire d’Innovation Moléculaire et Applications (LIMA), University of Strasbourg | University of Haute-Alsace | CNRS (UMR 7042), Equipe de Synthèse Organique et Molécules Bioactives (SYBIO), ECPM, 25 Rue Becquerel, 67000 Strasbourg, France; jeremy.schneider@live.fr (J.P.S.); philippe.compain@unistra.fr (P.C.); 3 Soft Matter Nanotechnology Lab, CIC biomaGUNE, Basque Research and Technology Alliance (BRTA), Paseo Miramón 182, 20014 Donostia-San Sebastián, Gipuzkoa, Spain; aangie.ramirez@gmail.com (M.d.l.A.R.); smoya@cicbiomagune.es (S.M.); 4 Associated with LENS, Via N. Carrara 1, 50019 Sesto Fiorentino, Italy

**Keywords:** multivalency, gold nanoparticles, iminosugars, enzyme inhibition, Jack bean α-mannosidase

## Abstract

Among carbohydrate-processing enzymes, Jack bean α-mannosidase (JBα-man) is the glycosidase with the best responsiveness to the multivalent presentation of iminosugar inhitopes. We report, in this work, the preparation of water dispersible gold nanoparticles simultaneously coated with the iminosugar deoxynojirimycin (DNJ) inhitope and simple monosaccharides (β-d-gluco- or α-d-mannosides). The display of DNJ at the gold surface has been modulated (i) by using an amphiphilic linker longer than the aliphatic chain used for the monosaccharides and (ii) by presenting the inhitope, not only in monomeric form, but also in a trimeric fashion through combination of a dendron approach with glyconanotechnology. The latter strategy resulted in a strong enhancement of the inhibitory activity towards JBα-man, with a *K_i_* in the nanomolar range (*K_i_* = 84 nM), i.e., more than three orders of magnitude higher than the monovalent reference compound.

## 1. Introduction

More than ten years have passed since the first example of a trivalent deoxynojirimycin (DNJ) derivative, that displayed a small, but quantifiable, inhibitory multivalent effect for Jack bean α-mannosidase (JBα-man), was reported [1]. Until then, the multivalency concept was considered an exclusive prerogative of lectin-carbohydrate interactions, and its application to carbohydrate-processing enzymes was almost completely unexplored, being considered extremely challenging, from a practical standpoint, and theoretically arguable. The refutation of such speculations was apparent in 2010, when a fullerene-based 12-valent DNJ compound showed binding enhancements towards JBα-man up to three orders of magnitude over the monovalent counterpart (inhibition constant *K_i_* = 0.15 µM vs. *K_i_* = 188 µM) [2].

After that, a plethora of different scaffolds have been employed for the multimerization of DNJ, ranging from small polyols, β-cyclodextrins, porphyrins, calixarenes, and cyclopeptoids to more complex micellar self-assembled glycopeptides and polymeric dextrans [3,4,5,6,7,8,9].

The largest binding enhancement ever reported for an enzyme inhibitor, so far, was obtained with a cyclopeptoid core, decorated with 36 DNJ units [10], which showed a nanomolar inhibition value (*K_i_* = 0.0011 µM). More importantly, the outstanding multivalent effect observed with this 36-valent cluster has been fully rationalized thanks to the recent achievement of the first high resolution crystal structure of its complex with JBα-man [11].

JBα-man enzyme (220 kDa) is a homodimer (LH)_2_ bearing two active sites. The crystal structure revealed the formation of a 2:1 JBα-man:inhibitor complex, in which four DNJ inhitopes bind the four active sites of two homodimers [11]. The reported X-ray data of the JBα-man:inhibitor complex did not show any binding to secondary binding sites. Conversely, the observed multivalent effect was rationalized by invoking a chelate binding mode [11]. Through TEM studies, a different homodimer aggregation featuring an S-shape arrangement was proposed for JBα-man in the presence of a tetravalent pyrrolidine iminosugar 1,4-dideoxy-1,4-imino-d-arabinitol (DAB-1) inhibitor [12]. More generally, these data revealed that the binding modes of JBα-man with iminosugar-based multivalent inhibitors strongly depend not only on the bioactive inhitope but also on the size and shape of the prepared multivalent architectures. Surprisingly, glyco-coated multivalent clusters also displayed some inhibition power on glycosidases instead of being hydrolyzed, as shown by the 12-valent mannosylated fullerene (*K_i_* of 320 µM for JBα-man) [13], glyconanodiamonds (*K_i_* between 222 and 517 µM against JBα-man) [14], and perglucosylated or permannosylated cyclodextrins (*IC_50_* of 32–132 µM against *Saccharomyces cerevisiae* α-glucosidase) [15] probably arising from a lectin-like behavior in those clusters.

In this context, we envisaged that glyco-gold nanoparticles (AuGNPs), decorated with different loading and spatial presentation of DNJ inhitopes, could represent a versatile and useful molecular tool for further mechanistic studies.

AuGNPs, as prepared in our laboratory, are water-dispersible and biocompatible gold nanoparticles coated with a 3D polyvalent carbohydrate shell, having a globular shape, chemically defined composition, and exceptionally small core size (about 2 nm) [16]. It is possible to simultaneously attach more than one kind of ligand onto the gold core (multifunctionality) and to modulate the ligand presentation on the metal surface in order to obtain multifunctional materials for application in nanomedicine (targeting, drug delivery, pathway inhibition, etc.) [17].

Despite the large number of different scaffolds employed for the DNJ multimerization, only three examples of nanoparticles (two inorganic [18,19] and one peptide-based [20]), decorated with this iminosugar, have been reported for different applications, to the best of our knowledge.

Some of us recently found that iminosugar-decorated AuGNPs retain inhibitory activity towards commercially available glycosidases [21]. We also reported that AuGNPs, decorated with DAB-1 iminosugar, were able to efficiently inhibit a therapeutically relevant enzyme [22]. Therein, a clickable iminosugar dendron approach [23] was combined with glyco-gold nanotechnology in order to obtain multivalent DAB-1 nanosystems with a denser iminosugar shell.

**Figure 1:**
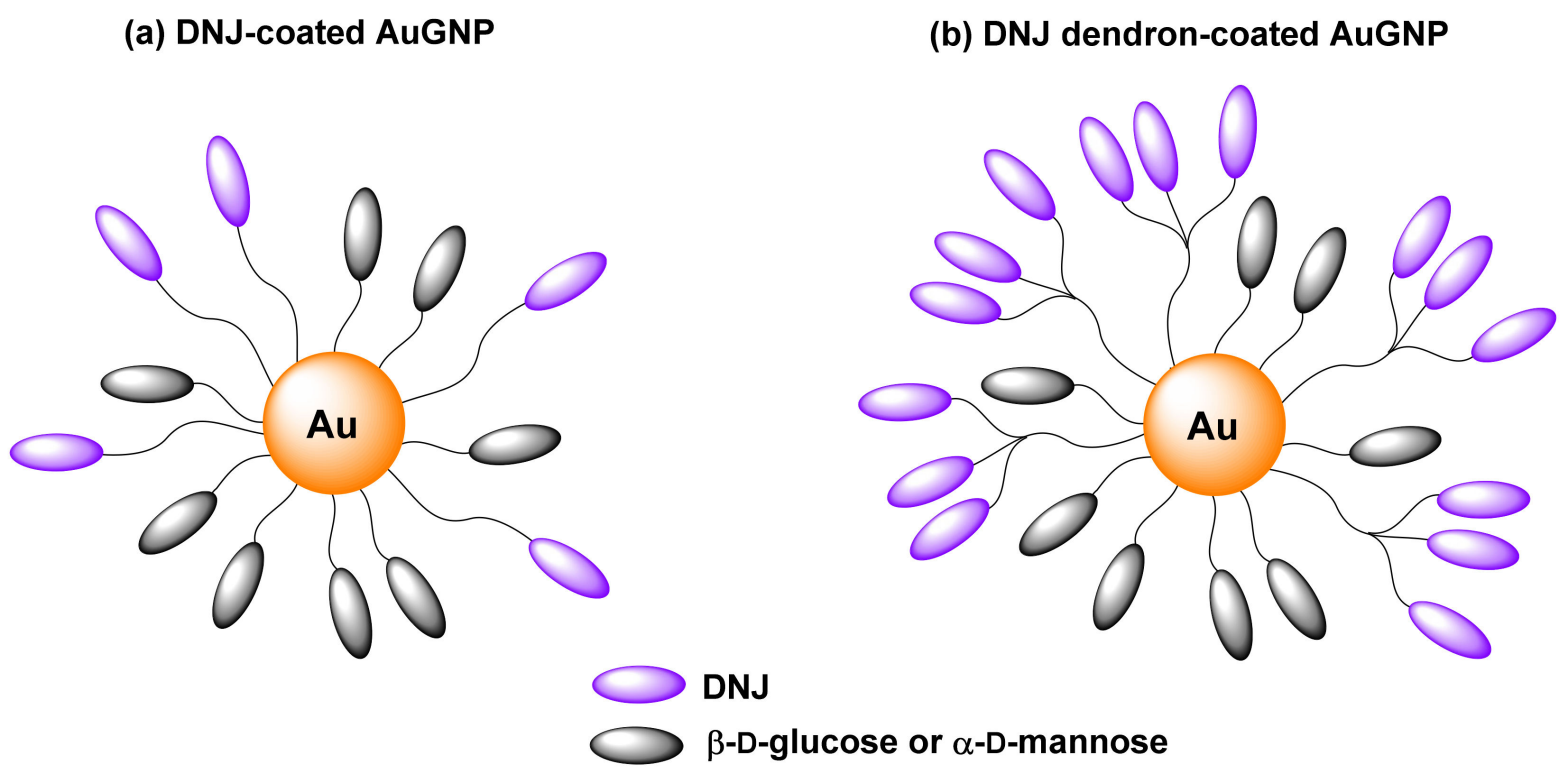
(**a**) Multivalent presentation of DNJ, using glyco-gold nanoparticles as scaffold and (**b**) the combination of glyco-gold nanotechnology with the iminosugar dendron approach.

In the present work, we report our results, obtained applying this methodology to the preparation and characterization of AuGNPs decorated with simple monosaccharides, heterovalent DNJ-based ligands, either linear or dendritic (Figure 1), and their biological evaluation towards JBα-man.

## 2. Results and Discussion

### 2.1. Preparation of Ligands and Gold Nanoparticles

**Figure 2:**
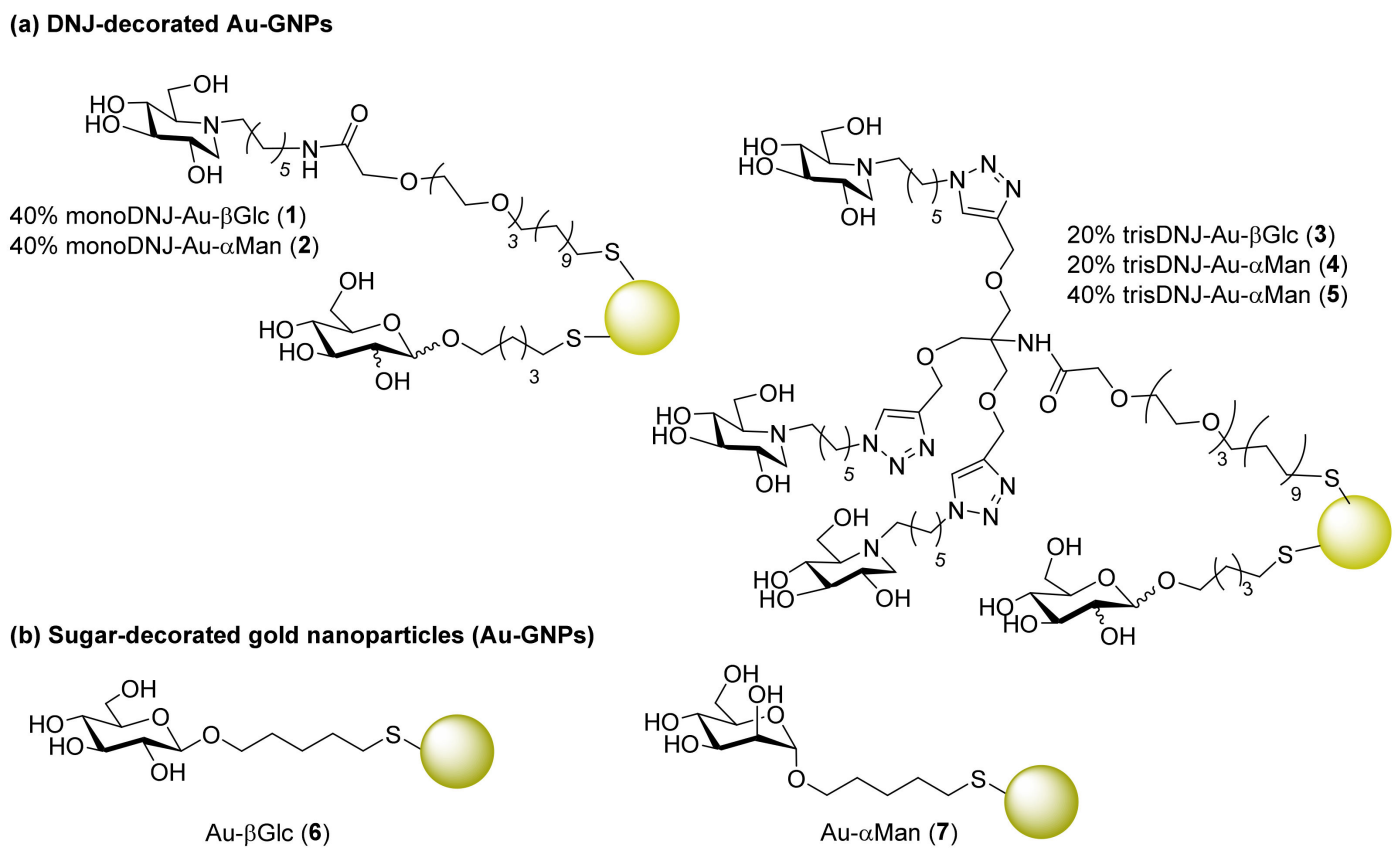
(**a**) Schematic representation of the multivalent glyco-gold nanoparticles coated with the iminosugar DNJ derivatives and monosaccharides (β-D-glucose or α-D-mannose derivatives) prepared in this work. (**b**) Nanoparticles coated with the monosaccharides (control systems).

The gold nanoparticles (**1**–**5**) prepared in this work (Figure 2) are constituted by an approximately 2 nm gold core coated with two components: simple monosaccharides (d-glucose or d-mannose) and DNJ derivatives. We played on the nature of the inner monosaccharide and varied the density of DNJ heads on the periphery of the nanoparticles. Nanoparticles **6** and **7**, in Figure 2, bearing 100% monosaccharides with a short and linear five carbons (C_5_) aliphatic linker, were prepared as control systems. For AuGNPs **1**–**5**, the carbohydrate moieties guarantee their water dispersibility, while the water soluble and flexible amphiphilic linker was designed to protrude the DNJ heads from the glyco-coated nanoparticle core. In addition, the *N*-C_6_ aliphatic linker of the active component DNJ is taking part in the interaction with the catalytic site entry [11,24]. This strategy of ligand presentation was based on our previous experience with similar constructs [21,22,25].

**Scheme 1:**
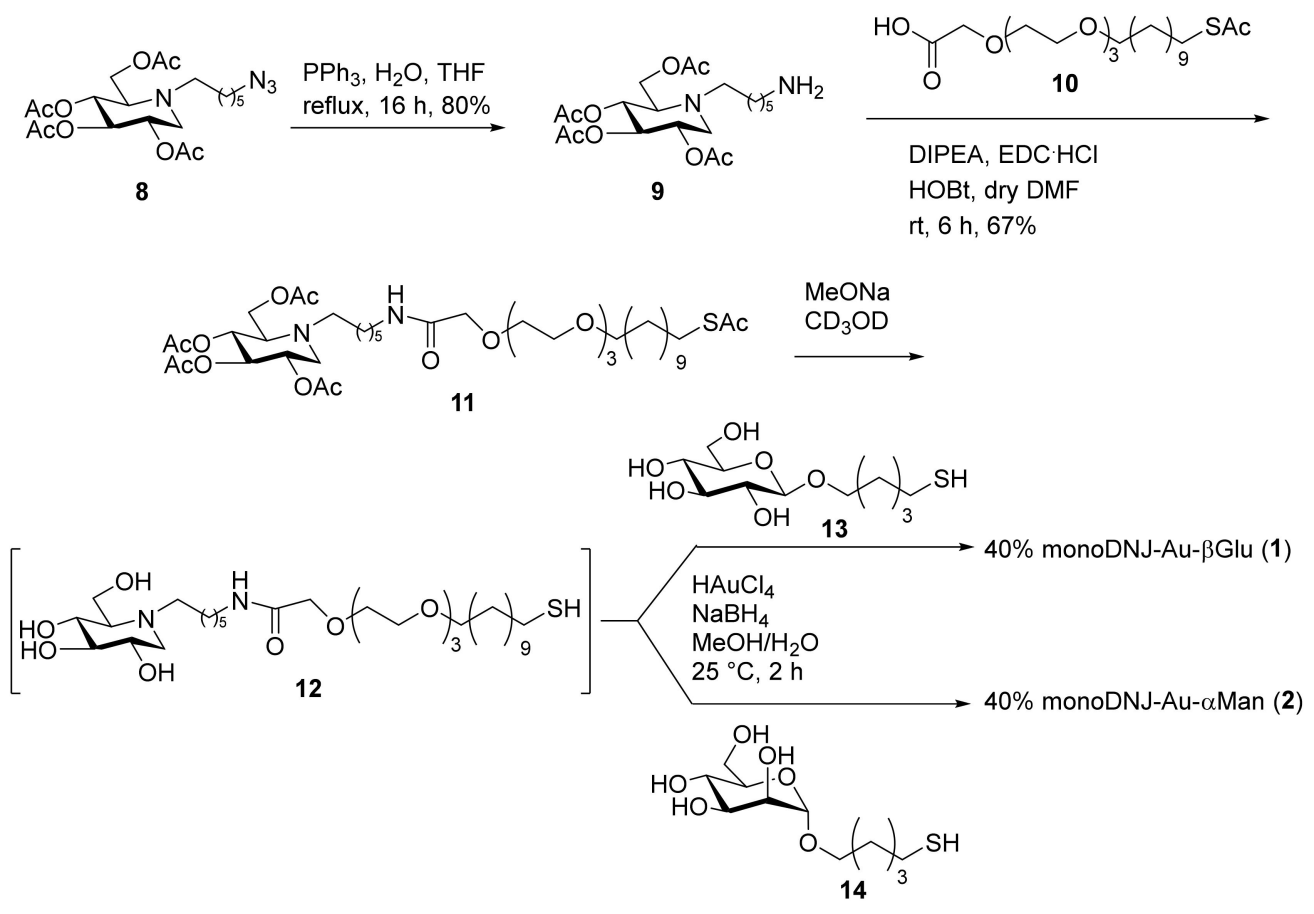
Synthesis of the glyco-gold nanoparticles **1** and **2** decorated with the DNJ monovalent ligand **12**.

In particular, AuGNPs **6** and **7** were prepared employing, as exclusive components, 5-mercaptopent-1-yl β-d-glucopyranoside (βGlcC_5_SH, **13**) or 5-mercaptopent-1-yl α-d-mannopyranoside (αManC_5_SH, **14**), following well established protocols [25]. AuGNPs **1** and **2** were prepared by employing the monovalent DNJ derivative **12** as the active component and **13** or **14**, respectively, as the inner component (Scheme 1).

The preparation of the monovalent DNJ derivative **12** is described in Scheme 1. The peracetylated azido-functionalized DNJ **8** [26] was reduced to the corresponding amino derivative **9** through a standard Staudinger reaction (PPh_3_, H_2_O, THF, reflux for 16 h) in 80% yield. The same reaction performed with polymer-bound PPh_3_ [22,27], and refluxing in THF/H_2_O, required longer times and provided **9** in lower yields (48%).

The aminoalkyl DNJ **9** was then coupled to the thiol-protected carboxylic acid **10**, prepared, as reported in the literature [21], using 1-ethyl-3-(3-dimethylaminopropyl) carbodiimide (EDC) and 1-hydroxybenzotriazole (HOBt) in dry DMF. The amide **11** was obtained in 67% yield after purification by flash column chromatography (FCC). Removal of the acetyl groups of **11** was performed under Zemplén conditions (MeONa) but using CD_3_OD, instead of MeOH, as solvent to follow the reaction via ^1^H NMR. Once compound **12** was formed, it was directly used for the next step, avoiding its oxidation to disulfide. AuGNPs **1** and **2** were prepared by mixing **12** with βGlcC_5_SH (**13**) or αManC_5_SH (**14**), respectively, in the appropriate ratio (40/60) and then adding an Au(III) solution (the total amount of thiols being 3 equiv. with respect to gold) and the reducing agent NaBH_4_ in excess (Scheme 1). The proportion of the ligands on the gold surface was evaluated by integrating diagnostic signals in the ^1^H NMR spectrum of the initial mixture and in the ^1^H NMR spectrum of the supernatant after AuGNPs formation (see Supplementary Materials for ^1^H NMR spectra). The 40/60 active/inner component ratio allowed to preserve nanoparticle dispersibility in water [22]. After shaking for 2 h at room temperature, the supernatant was removed, and the nanoparticles were washed with methanol. The residue was dispersed in milliQ water, purified by dialysis, and freeze-dried. These nanoparticles were re-dispersed in water after the freeze-drying process, and no flocculation was observed even after several months. Full characterization with different techniques (see below) was carried out before enzymatic evaluation.

**Scheme 2:**
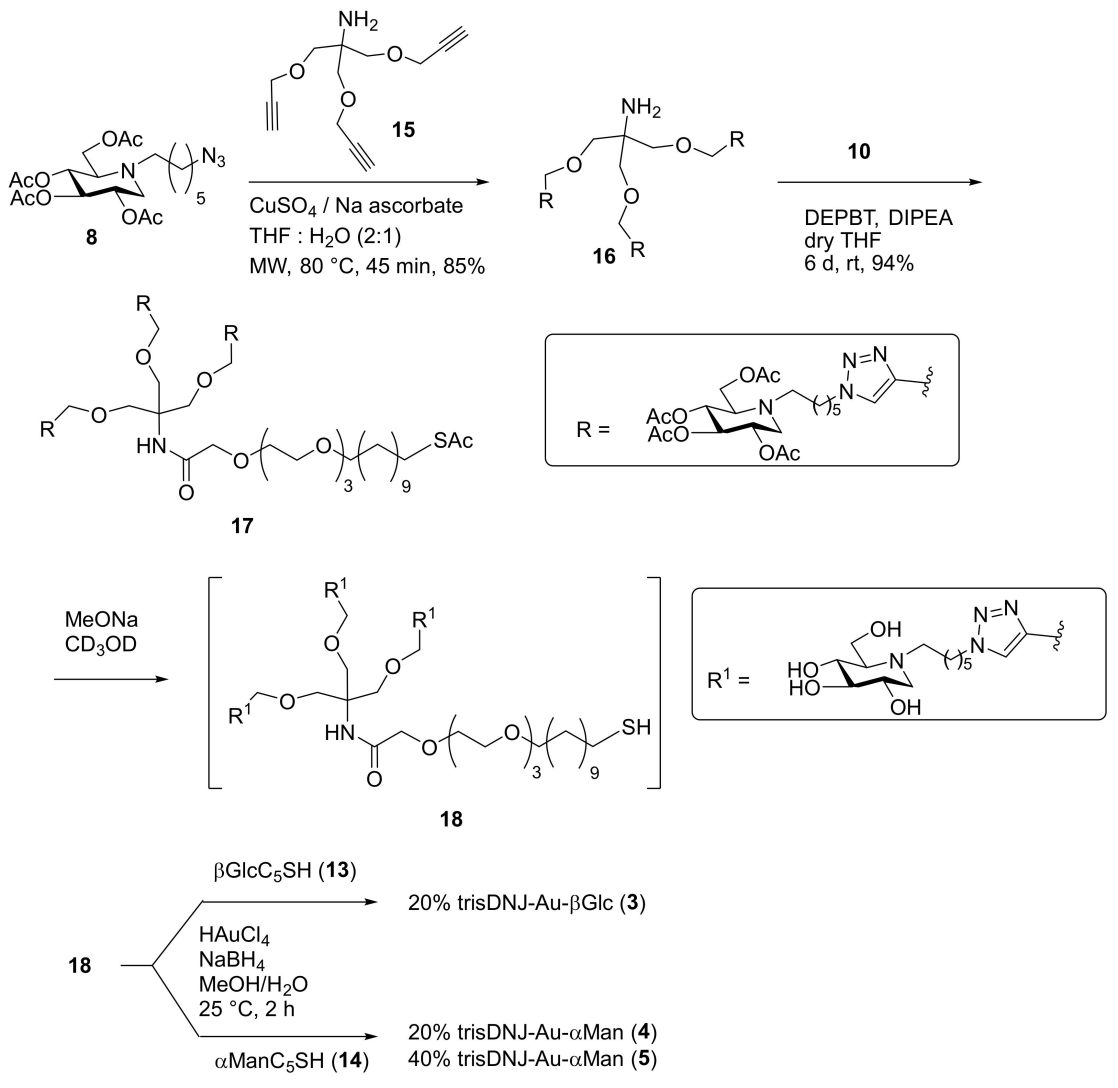
Synthesis of the trivalent ligand 18 and preparation of AuGNPs **3**–**5**.

To further increase the DNJ loading onto AuGNPs, we combined this strategy with the dendron approach by preparing the trivalent DNJ derivative **18** (Scheme 2). The peracetylated azido-functionalized DNJ **8** was first reacted with the trialkyne **15** [28] to afford the dendron 16 in 85% yield through copper (I) catalyzed azide alkyne cycloaddition (CuAAC) reaction [29,30,31]. Subsequently, introduction of the linker was achieved by coupling the carboxylic acid **10** with **16** in the presence of 3-(diethoxyphosphoryloxy)-1,2,3-benzotriazin-4(3H)-one (DEPBT) as coupling agent affording the final ligand **17** in an excellent 94% yield. In analogy with the incorporation of the monovalent ligand **12** onto AuGNPs, trivalent thiol **18** was generated in situ, and its density on the nanoparticles was modulated with βGlcC5SH (**13**) (to yield AuGNPs **3**) or αManC5SH (**14**) (to yield AuGNPs **4** and **5**), as described above. The mannose-based AuGNPs **4** and **5** shared the same inner component but in different percentages (80% in **4**, 60% in **5**).

**Figure 3:**
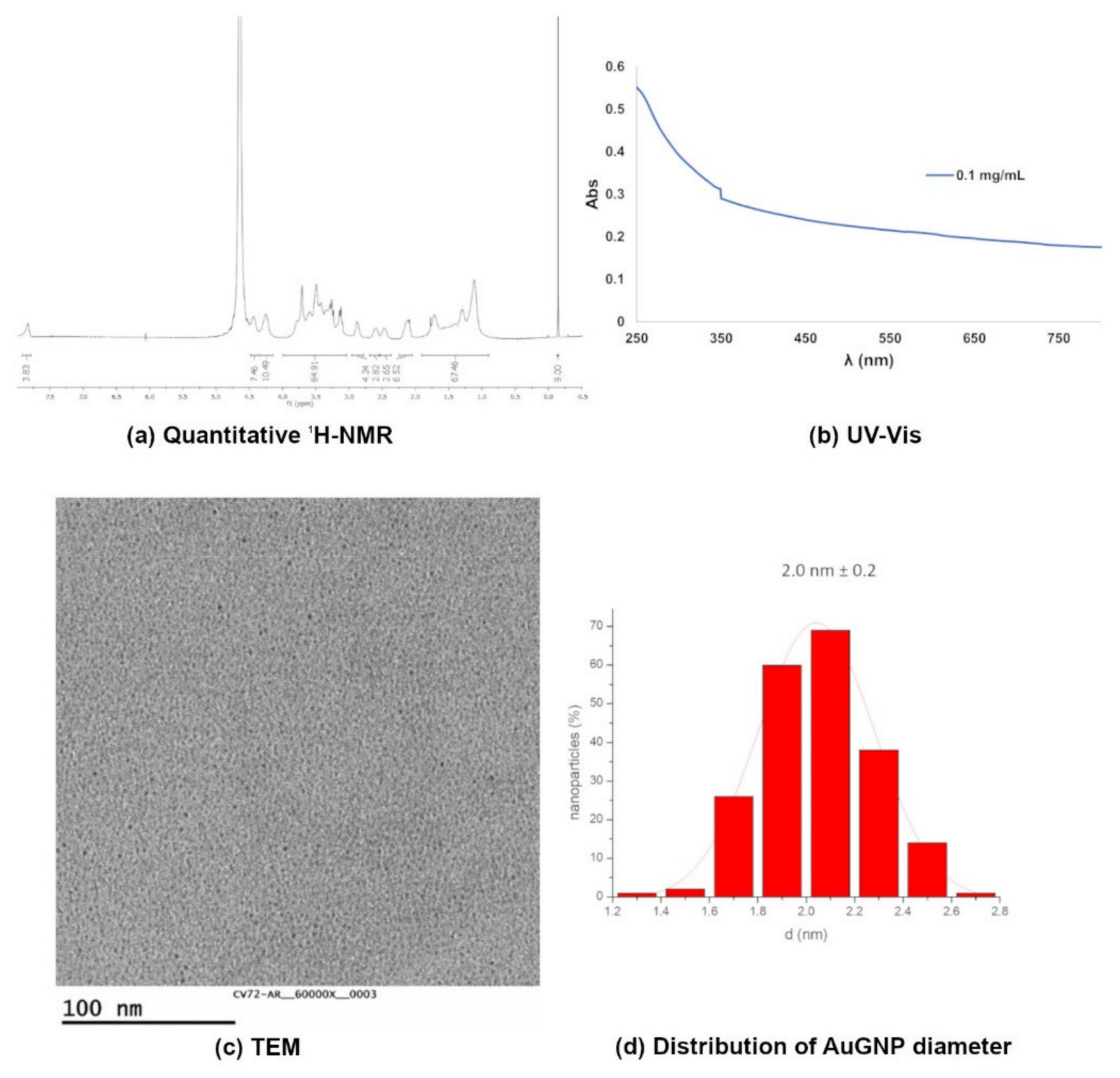
Characterization of AuGNP **3** (20% trisDNJ-Au-βGlc): (**a**) quantitative ^1^H NMR (D_2_O) spectrum (400 MHz); (**b**) UV/Vis spectrum; (**c**) TEM micrograph in H_2_O; (**d**) size-distribution histogram, obtained by measuring 300 nanoparticles (average diameter 2.0 ± 0.2 nm).

AuGNPs **1**–**5** were subjected to quantitative ^1^H NMR (qNMR) analysis in D_2_O with 3-(trimethylsilyl)propionic-*2,2,3,3-d_4_* acid (TSP-d_4_) as an internal standard, following established protocols [21,32,33]. As an example, Figure 3a shows the qNMR for AuGNP **3**. In this way, it was possible to quantify the DNJ amount on the nanoparticle by integration of a diagnostic signal (see Supplementary Materials and Table S1) with respect to the internal standard. An average gold core size in 1.6–2.1 nm range was determined by TEM imaging for the AuGNPs (Figure 3c,d) [25,34]. This size was also confirmed by UV-Vis spectra, which did not show an absorption maximum at around 520 nm, typical of gold nanoparticles with a bigger core diameter [35] (Figure 3b).

### 2.2. Biological Evaluation

**Figure 4:**
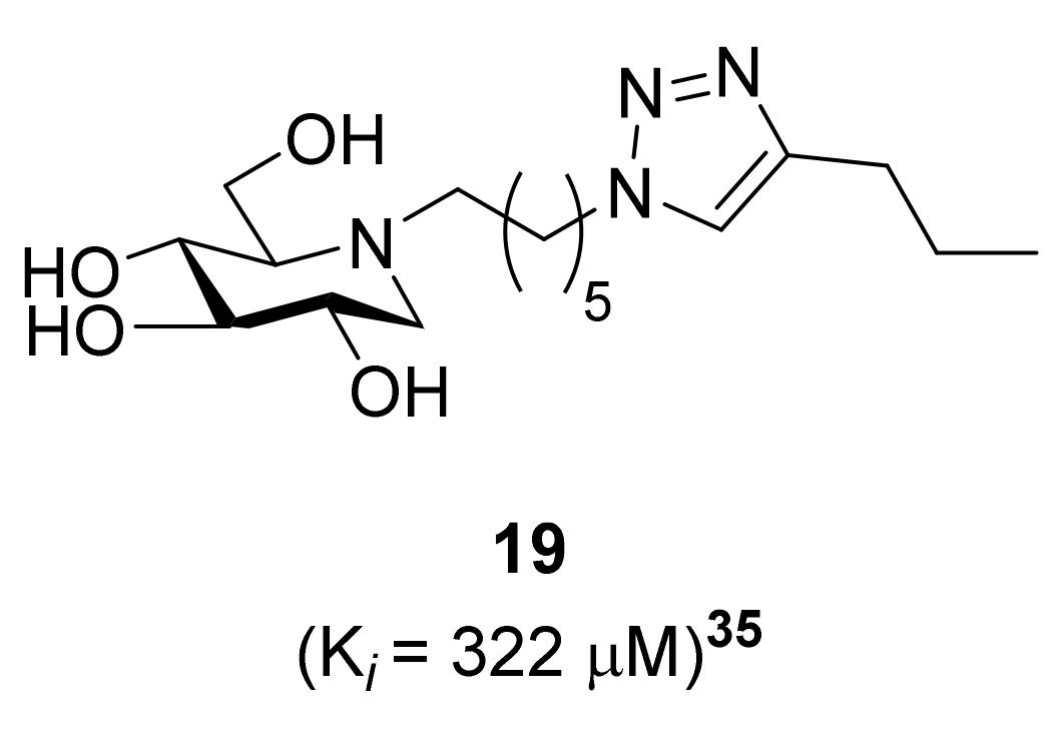
DNJ derivative chosen as the monovalent reference to evaluate the relative potency of the AuGNPs synthesized in this work.

The DNJ-coated AuGNPs **1**–**5** were evaluated against Jack bean α-mannosidase (JBα-man), the glycosidase which showed the largest response to multivalent inhibitor presentation to date [3,4,5,6,7,8,9]. The inhibition mode and constants (*K_i_*) were obtained from Dixon or Lineweaver Burk plots for a scale of inhibitor concentrations reflecting the DNJ concentration directly. The inhibition potency, relative to the corresponding monovalent reference **19** [36] (Figure 4), directly gives the relative potency per inhitope (*rpn*) (Table 1). 

The whole set of newly prepared heterovalent AuGNPs **1**–**5** showed good to excellent inhibitory activity towards JBα-man, with *K_i_* values in the low micromolar range to the nanomolar one. All behaved as competitive inhibitors (see Supplementary Materials). Conversely, β-d-gluco- and α-d-mannoside decorated AuGNPs **6** and **7** did not inhibit the enzyme at 0.5 mg mL^−1^ (This concentration was a good compromise to allow spectrophotometric measurements due to the brown color of AuGNP solutions. The 12-valent mannosylated fullerene from Vincent and Nierengarten et al. (see ref [13]) was active at the hundreds of µM). However, trimerization of DNJ at the gold surface had a major impact: it strongly enhances the affinity towards JBα-man (**3** vs. **1**, **4** vs. **2**, Table 1), with the affinity enhancement per inhitope increasing up to 1840 for AuGNP **4**. A further enhancement of the inhibitory properties was reached upon increasing the loading of the trivalent DNJ derivative **18** onto the gold surface from 20% to 40%, with a *K_i_* at 84 nM and an *rpn* value of 3833 observed for AuGNP **5** (Table 1). 

Noticeably, the dendron strategy is a double-edged sword. On the one hand, the intrinsic hindrance of the iminosugar tripod seems to prevent loading as high as linear arms (It is worth noting that for a same percentage loading of d-mannose of 60%, the DNJ concentration on AuGNP **5** is not 3 times higher than that of AuGNP **2**, suggesting that the total loading on the particle is lower than for AuGNP **2**. However, the ratio between iminosugar DNJ derivatives and monosaccharides still reflects the composition of the preparation mixture, as attested by ^1^H-NMR spectra recorded before and after (supernatant) the formation of the AuGNPs (see Supplementary Materials)). On the second hand, although the DNJ concentration is almost identical (503 vs. 467 for AuGNP **2** and **5**), the inhibition constant of AuGNP **5** is more than 100 times better (Table 1 and Table S1). Despite this lower coating, the multivalent effect, as judged by the *rpn*, is remarkably increased by two orders of magnitude. The spatial distribution of DNJ at the AuGNP **5**, as obtained through the dendron strategy, is highly beneficial to the enzymatic affinity. AuGNPs thus represent an opportunity to compare multivalent systems, having similar concentrations in DNJ inhitopes, but with different local densities. In DNJ dendron-coated AuGNPs, the higher local concentration may favor the bind-and-recapture of the reversible inhibitor heads. 

## 3. Materials and Methods

### 3.1. General Experimental Procedures for the Syntheses

Commercial reagents were used as received. All reactions were carried out under magnetic stirring and monitored by TLC on 0.25 mm silica gel plates (Merck F254). Column chromatographies were carried out on Silica Gel 60 (32–63 μm) or on silica gel (230–400 mesh, Merck, Darmstadt, Germany). Yields refer to spectroscopically and analytically pure compounds unless otherwise stated. ^1^H NMR spectra were recorded on a Varian Gemini 200 MHz, a Varian Mercury 400 MHz, or on a Varian INOVA 400 MHz instrument at 25 °C. Additionally, ^13^C NMR spectra were recorded on a Varian Gemini 200 MHz or on a Varian Mercury 400 MHz instrument. Chemical shifts are reported relative to CDCl_3_ (^13^C: δ = 77.0 ppm), or to CD_3_OD (^13^C: δ = 49.0 ppm). The following abbreviations were used to designate multiplicities: s = singlet, d = doublet, t = triplet, m = multiplet, br = broad, and dd = double-doublet. Integrals are in accordance with assignments, coupling constants are given in Hz. For detailed peak assignments, 2D spectra were measured (COSY, HSQC). IR spectra were recorded with a IRAffinity-1S SHIMADZU system spectrophotometer. ESI-MS spectra were recorded with a Thermo Scientific™ LCQ fleet ion trap mass spectrometer. Elemental analyses were performed with a Thermo Finnigan FLASH EA 1112 CHN/S analyzer. Optical rotation measurements were performed on a JASCO DIP-370 polarimeter. TEM analysis was performed with a LaB6-TEM of type JEOL JEM-1400PLUS (40 kV–120 kV, HC pole piece) equipped with a GATAN US1000 CCD camera (2 k × 2 k). 

#### 3.1.1. Synthesis of Monovalent DNJ Derivative **9**

To a solution of **8** (36 mg, 79 μmol) in 1.5 mL of THF 4.6 mL and 3 μL of H_2_O (145 μmol), PPh_3_ (25 mg, 87 μmol) was added. The reaction mixture was then refluxed for 16 h, until a TLC analysis (CH_2_Cl_2_/MeOH 10: 1) showed the disappereance of the starting material (*R*f = 0.91) and the formation of a new product (*R*f = 0.10). The solvent was removed under reduced pressure and the crude was purified by FCC (from CH_2_Cl_2_/MeOH 10:1 to CH_2_Cl_2_/MeOH/NH_3_ 5:1:0.2) affording pure **9** (25 mg, 58.1 μmol, *R*f = 0.10 in CH_2_Cl_2_/MeOH 10:1) as a colourless oil in 80% yield. ${\left[\alpha \right]}_{D}^{22}$ = 4.75 (c = 0.59 in CHCl_3_); ^1^H-NMR (400 MHz, CDCl_3_): δ = 5.05 (dt, *J* = 17.1, 9.2 Hz, 2H, H-3, H-4), 4.94 (td, *J* = 10.0, 5.0 Hz, 1H, H-2), 4.19–4.09 (m, 2H, H-6), 3.18 (dd, *J* = 11.5, 5.1 Hz, 1H, Ha-1), 2.75–2.68 (m, 1H, Ha-1′), 2.62 (dt, *J* = 11.6, 2.5 Hz, 1H, H-5), 2.57–2.50 (m, 1H, Hb-1′), 2.43 (bs, 2H, H-6′), 2.30 (t, *J* = 10.5 Hz, 1H, Hb-1), 2.06 (s, 3H, OC*H*_3_), 2.01 (s, 6H, OC*H*_3_), 2.00 (s, 3H, OC*H*_3_), 1.48–1.22 (m, 8H, H-2′, H-3′, H-4′, H-5′) ppm; ^13^C-NMR (100 MHz, CDCl_3_): δ = 171.0–169.9 (4C, O*C*OCH_3_), 74.8 (C-2), 69.7 (C-4), 69.6 (C-3), 61.7 (C-5), 59.7 (C-6), 53.0 (C-1), 51.8 (C-1′), 27.1–26.8 (4C, from C2′ to C5′), 24.9 (C-6′), 21.0–20.8 (4C, CO*C*H_3_) ppm; MS (ESI): *m*/*z* 431.04 (100, H^+^). IR (CDCl_3_): $\tilde{\nu }$ = 2935, 2860, 2255, 1744, 1664, 1601, 1508, 1369, 1234, 1061, 1032 cm^−1^. Elemental analysis (%) for C_20_H_34_N_2_O_8_ (430.49): calcd C, 55.80; H, 7.96; N, 6.51; found: C, 56.34; H, 7.57; N, 6.90.

#### 3.1.2. Synthesis of Monovalent DNJ Based Ligand **11**

A solution of EDC^.^HCl (1-Ethyl-3-(3-dimethylaminopropyl) carbodimide hydrochloride (18.2 mg, 95 µmol), 1-hydroxybenzotriazole (HOBt, 11.7 mg, 87 µmol), and **10** (25.9 mg, 59 µmol) in dry DMF (0.2 mL) was left stirring for 10 min. and then added to a solution of DNJ derivative **9** (17.0 mg, 40 µmol) and *N*,*N*-diisopropylethylamine (19 µL, 107 µmol) in DMF (0.9 mL). The reaction mixture was left stirring at room temperature, under nitrogen atmosphere, for 6 h, then diluted with AcOEt (10 mL) and washed with H_2_O (2 × 3 mL). The organic layer was then washed with a saturated solution of NaHCO_3_ (1 × 3 mL) and brine (1 × 4 mL), dried over anhydrous Na_2_SO_4_ and concentrated under vacuum. The crude was purified by column chromatography (DCM/MeOH from 30:1 to 10:1) affording 22 mg of **11** (26 µmol, 67% yield). *R*f = 0.32 (CH_2_Cl_2_/MeOH 10:1). ${\left[\alpha \right]}_{D}^{26}$ = 3.33 (c = 0.69 in CHCl_3_). ^1^H-NMR (400 MHz, CDCl_3_): δ = 5.06 (dt, *J* = 9.2, 2.8 Hz, 2H, H-3, H-4), 4.99–4.92 (m, 1H, H-2), 4.17–4.13 (m, 2H, H-6), 3.98 (s, 2H, C*H*_2_ = CO), 3.69–3.56 (m, 12H), 3.44 (t, *J* = 6.8 Hz, 2H), 3.29–3.16 (m, 3H, H-6′, Ha-1), 2.85 (t, *J* = 3.6 Hz, 2H, C*H*_2_SAc), 2.77–2.69 (m, 1H, Ha-1′), 2.64–2.61 (m, 1H, H-5), 2.58–2.52 (m, 1H, Hb-1′), 2.32 (bs, 4H, SCOC*H*_3_, Hb-1), 2.08 (s, 3H, OC*H*_3_), 2.02 (s, 6H, OC*H*_3_), 2.01 (s, 3H, OC*H*_3_), 1.59–1.45 (m, 4H), 1.36–1.25 (m, 22H) ppm; ^13^C-NMR (50 MHz, CDCl_3_): δ = 196.0 (S*C*OCH_3_), 170.8-170.0 (4C, −O*C*OCH_3_), 169.7 (*C*ONH), 74.7 (C-2), 71.6–70.0 (8C), 69.5 (C-4), 69.4 (d, C-3), 61.5 (C-5), 59.5 (C-6), 52.9 (C-1), 51.7 (C-1′), 38.7 (C-6′), 30.6 (SCO*C*H_3_), 29.6–28.8 (11C), 26.9–26.1 (3C, C-2′, C-3′, C-4′), 20.8–20.6 (4C, CO*C*H_3_) ppm; MS (ESI): *m*/*z* 871.35 (100, M+Na^+^). IR (CDCl_3_): $\tilde{\nu }$ = 3005, 2929, 2856, 1744, 1673, 1540, 1466, 1370, 1237, 1107, 1033 cm^−1^; Elemental analysis (%) for C_41_H_72_N_2_O_14_S (849.08): calcd C, 58.00; H, 8.55; N, 3.30; found: C, 56.44; H, 7.90; N, 2.39.

#### 3.1.3. Synthesis of Trivalent DNJ Derivative **16**

To a solution of **8** (63 mg, 138 μmol) in 3 mL of THF/H_2_O = 2:1 CuSO_4_ (2.1 mg, 13 μmol), sodium ascorbate (5 mg, 25 μmol) and **15** [28] (9.9 mg, 42 μmol) were added. The reaction mixture was stirred in microwave at 80 °C for 45 min, until a TLC analysis (CH_2_Cl_2_/MeOH 10: 1) showed the disappearance of the starting material (*R*f = 0.46) and the formation of a new product (*R*f = 0.00). The solvent was removed under reduced pressure and the crude was purified by FCC (CH_2_Cl_2_/MeOH/NH_3_ from 30:1 to 5: 1: 0.1) affording pure **16** (57 mg, 35.4 μmol, *R*f = 0.33 in CH_2_Cl_2_/MeOH/NH_3_ 10:1:0.1) as a pale yellow oil, in 85% yield. ${\left[\alpha \right]}_{D}^{22}$ = 7.47 (c = 0.91 in CHCl_3_). ^1^H-NMR (400 MHz, CDCl_3_): δ = 7.56 (br s, 3H, triazole), 5.09–5.01 (m, 6H, H-3, H-4), 4.97–4.91 (m, 3H, H-2), 4.60 (s, 6H, H-7′), 4.34 (t, *J* = 7.2 Hz, 6H, H-6′), 4.18–4.10 (m, 6H, H-6), 3.44 (s, 6H, H-8′), 3.18 (dd, *J* = 11.4, 5.0 Hz, 3 H, Ha-1), 2.76-2.69 (m, 3H, Ha-1′), 2.63–2.60 (m, 3H, H-5), 2.56–2.50 (m, 3H, Hb-1′), 2.29 (t, *J* = 10.8 Hz, 3H, Hb-1), 2.07 (s, 9H, OC*H*_3_), 2.02–2.01 (m, 27H, OC*H*_3_), 1.94–1.84 (m, 6H, H-5′), 1.45–1.25 (m, 18H, H-2′, H-3′, H-4′) ppm; ^13^C-NMR (100 MHz, CDCl_3_): δ = 170.9-169.9 (12C, O*C*OCH_3_), 145.2 (3C, triazole), 122.5 (3C, triazole), 74.8 (3C, C-2), 72.4 (3C, C-8′), 69.7 (3C, C-4), 69.6 (3C, C-3), 65.2 (3C, C-7′), 61.9 (3C, C-5), 59.8 (3C, C-6), 56.3 (NH_2_*C*(CH_2_O-)_3_), 53.0 (3C, C-1), NH_2_*C*(CH_2_O-)_3_), 51.7 (3C, C-1′), 50.3 (3C, C-6′), 30.4 (3C, C-5′), 26.8 (3C, C-2′), 26.6 (3C, C-3′), 25.1 (3C, C-4′), 21.0–20.8 (12C, CO*C*H_3_) ppm; MS (ESI): *m*/*z* 1626.58 (100, Na^+^); IR (CDCl3): $\tilde{\nu }$ = 3690, 3606, 2937, 2862, 2257, 1745, 1602, 1438, 1370, 1234, 1097, 1052, 1032 cm^−1^. Elemental analysis (%) for C_73_H_113_N_13_O_27_ (1604.75): calcd C, 54.64; H, 7.10; N, 11.35; found: C, 54.60; H, 7.35; N, 10.37.

#### 3.1.4. Synthesis of Trivalent DNJ Based Ligand **17**

To a solution of compound **16** (56.8 mg, 35.4 µmol) in dry THF (570 μL) at 0 °C, 3-(diethoxyphosphoryloxy)-1,2,3-benzotriazin-4(3H)-one (DEPBT 21.2 mg, 70.8 μmol) and *N*,*N*-diisopropylethylamine (DIPEA 12.0 μL, 70.8 μmol) were added. The mixture was stirred at 25 °C for 15 min under a nitrogen atmosphere, then a solution of **10** (30.0 mg, 21.6 μmol) in dry THF (225 μL) was added and the reaction mixture was stirred at room temperature for 6 days, until a TLC analysis (CH_2_Cl_2_/MeOH 6:1) showed the formation of a new product (*R*f = 0.7). The reaction mixture was diluted with AcOEt (5 mL), washed with NH_4_Cl (2 × 3 mL), NaHCO_3_ (2 × 3 mL) and H_2_O (2 × 3 mL), dried over anhydrous Na_2_SO_4_ and concentrated under vacuum. Purification through gradient column chromatography (CH_2_Cl_2_/MeOH from 20:1 to 10:1) afforded pure **17** (61 mg, 30.1 μmol) as a yellow oil, in 94% yield. ${\left[\alpha \right]}_{D}^{18}$ = 5.29 (c = 0.87 in CHCl_3_). ^1^H-NMR (400 MHz, CDCl_3_): δ = 7.54 (br s, 3H, triazole), 6.81 (s, 1H, CON*H*), 5.06–4.98 (m, 6H, H-3, H-4), 4.94–4.88 (m, 3H, H-2), 4.57 (s, 6H, H-7′), 4.31 (t, *J* = 7.2 Hz, 6H, H-6′), 4.15–4.07 (m, 6H, H-6), 3.84 (s, 2H), 3.77 (s, 6H, H-8′), 3.63–3.51 (m, 12 H), 3.39 (t, *J* = 6.8 Hz, 2H), 3.15 (dd, *J* = 11.5, 5.1 Hz, 3H, Ha-1), 2.82 (t, *J* = 7.4 Hz, 2H), 2.74–2.67 (m, 3H, Ha-1′), 2.60–2.58 (m, 3H, H-5), 2.53–2-47 (m, 3H, Hb-1′), 2.28–2.24 (m, 6H, SCOC*H*_3_, Hb-1), 2.03 (s, 9H, OC*H*_3_), 1.99 (s, 18H, OC*H*_3_), 1.98 (s, 9H, OC*H*_3_), 1.91–1.84 (m, 6H, H-5′), 1.56–1.48 (m, 4H), 1.33–1.22 (m, 32H) ppm; ^13^C-NMR (50 MHz, CDCl_3_): δ = 196.0 (S*C*OCH_3_), 170.9–169.7 (12C, O*C*OCH_3_), 169.7 (*C*ONH), 144.9 (3C, triazole), 122.6 (3C, triazole), 74.8 (3C, C-2), 71.6–70.1 (8C), 69.6 (3C, C-4), 69.5 (3C, C-3), 68.9 (3C, C-8′), 65.1 (3C, C-7′), 61.8 (3C, C-5), 59.6 (4C, C-6, NH*C*(CH_2_O-)_3_), 53.0 (3C, C-1), 51.6 (3C, C-1′), 50.2 (3C, C-6′), 30.4 (CH_2_SCO*C*H_3_), 29.6–25.0 (22C), 20.9, 20.7 (12C, CO*C*H_3_) ppm; MS (ESI): *m*/*z* 1033.92 (100, (*m*/*z*) + 2Na^+^); IR (CDCl3): $\tilde{\nu }$ = 3022, 2932, 2858, 1744, 1675, 1525, 1369, 1220, 1098, 1032 cm^−1^. Elemental analysis (%) for C_94_H_151_N_13_O_33_S (2023.34): calcd C, 55.80; H, 7.52; N, 9.00; found: C, 55.76; H, 7.78; N, 8.22.

#### 3.1.5. General Procedure for the In Situ Preparation of Ligands **12** and **18**

To a solution of **11** or **17** in CD_3_OD (10 mg/mL), 30 equivalents of NaOMe were added, and the reaction mixture was left stirring for 2 h at 25 °C under nitrogen atmosphere. The complete disappearance of the starting material was attested via ^1^H NMR, and the crude, containing compounds **12** or **18**, respectively, was directly used for the preparation of AuGNPs.

#### 3.1.6. General Procedure for the Preparation of AuGNPs **1**–**5**

An aqueous solution of HAuCl_4_ (25 mM, 1 equiv.) was added to a 12 mM methanolic solution of a suitable mixture of thiol-ending monosaccharide and DNJ ligands (3 equiv. overall). An aqueous solution of NaBH_4_ (1 M, 27 equiv.) was then added in four portions, with vigorous shaking. The black suspension formed was shaken for 2 h at 25 °C. The residue was washed several times with MeOH. In order to effectively separate the nanoparticles from the supernatant, centrifugation (12,000 rpm, 2 min) was performed. The residue was dissolved in a minimal volume of HPLC gradient grade water and purified by dialysis (SnakeSkin^®^ Pleated Dialysis Tubing, 10,000 MWCO and Slide-A-Lyzer^®^ 10K Dialysis Cassettes, 10,000 MWCO). DNJ-coated AuGNPs were obtained as a dark-brown powder after freeze-drying and characterized via ^1^H NMR, UV-Vis spectroscopy and TEM analysis (see Supplementary Materials). For the analysis of the ratio between the active component (DNJ-based ligand) and the inner component (monosaccharide ligand), ^1^H NMR spectra of the initial mixture and the supernatant, after AuGNP formation, were recorded. The DNJ loading on the AuGNPs was evaluated by quantitative NMR (qNMR) using 3-(trimethylsilyl)propionic-*2,2,3,3-d*_4_ acid, sodium salt (TSP-d_4_) as an internal standard in the D_2_O solution of the AuGNPs. The prepared AuGNPs can be stored at 4 °C for months while maintaining their biophysical properties.

##### Preparation of 40% monoDNJ-Au-βGlc **1**

A 1:1.5 mixture of thiol-ending **12** (4.5 mg, 2.65 μmol) and βGlcC_5_SH **13** (1.1 mg, 3.89 μmol) in CD_3_OD (1.1 mL) was used to obtain 0.35 mg of AuGNP **1**. TEM (average diameter): 1.8 ± 0.4 nm. Quantitative ^1^H-NMR (400 MHz, D_2_O containing 0.05 wt.% of 3-(trimethylsilyl)propionic-*2,2,3,3-d*_4_ acid, sodium salt as an internal standard): 0.15 mg of **1** were dissolved in 200 μL of D_2_O and 15 μL of D_2_O, containing 0.05 wt.% TSP, were added and 31 nmoles of DNJ conjugate were found. In the quantitative NMR (qNMR) a mediated value of the multiplet corresponding to Ha-1’ proton signal (δ = 2.84 ppm, 1H) of DNJ conjugate and the multiplet corresponding to H-6 (δ = 2.60-2.45 ppm, 2H) was selected for integration as it falls in a spectral region free of other signals. UV-Vis (H_2_O, 0.1 mg/mL): absence of a maximum band at around 520 nm.

##### Preparation of 40% monoDNJ-Au-αMan **2**

A 1:1.5 mixture of thiol-ending **12** (3.8 mg, 5.89 μmol) and αManC_5_SH **14** (2.5 mg, 8.84 μmol) in CD_3_OD (1.2 mL) was used to obtain 1.33 mg of AuGNPs **2**. TEM (average diameter): 2.1 ± 0.6 nm. Quantitative ^1^H-NMR (400 MHz, D_2_O containing 0.05 wt.% of 3-(trimethylsilyl)propionic-*2,2,3,3-d*_4_ acid, sodium salt as an internal standard): 0.60 mg of **2** were dissolved in 200 μL of D_2_O and 40 μL of D_2_O containing 0.05 wt.% TSP were added and 140 nmoles of DNJ conjugate were found. Significant peaks: δ = 2.85–2.95 (br signal, 1H, from DNJ conjugate) ppm. UV-Vis (H_2_O, 0.1 mg/mL): absence of a maximum band at around 520 nm. 

##### Preparation of 20% trisDNJ-Au-βGlc **3**

A 1:4 mixture of thiol-ending **18** (10.9 mg, 7.41 μmol) and βGlcC_5_SH **13** (8.4 mg, 29.64 μmol) in CD_3_OD (3.1 mL) was used to obtain 4.5 mg of AuGNPs **3**. TEM (average diameter): 2.1 ± 0.5 nm. Quantitative ^1^H-NMR (400 MHz, D_2_O containing 0.05 wt.% of 3-(trimethylsilyl)propionic-*2,2,3,3-d*_4_ acid, sodium salt as an internal standard): 0.60 mg of **3** were dissolved in 180 μL of D_2_O and 40 μL of D_2_O containing 0.05 wt.% TSP were added and 170 nmoles of DNJ conjugate were found. Significant peaks: δ = 7.83 (br s, 3H, triazole from DNJ derivative) ppm. UV-Vis (H_2_O, 0.1 mg/mL): absence of a maximum band at around 520 nm.

##### Preparation of 20% trisDNJ-Au-αMan **4**

A 1:4 mixture of thiol-ending **18** (10.9 mg, 7.41 μmol) and αManC_5_SH **14** (8.3 mg, 29.64 μmol) in CD_3_OD (3.1 mL) was used to obtain 3.7 mg of AuGNP **4**. TEM (average diameter): 2.0 ± 0.4 nm. Quantitative ^1^H-NMR (400 MHz, D_2_O containing 0.05 wt.% of 3-(trimethylsilyl)propionic-*2,2,3,3-d*_4_ acid, sodium salt as an internal standard): 0.60 mg of **4** were dissolved in 180 μL of D_2_O and 40 μL of D_2_O containing 0.05 wt.% TSP were added and 135 nmoles of DNJ conjugate were found. Significant peaks: δ = 7.84 (br s, 3H, triazole from DNJ derivative) ppm. UV-Vis (H_2_O, 0.1 mg/mL): absence of a maximum band at around 520 nm.

##### Preparation of 40% trisDNJ-Au-αMan **5**

A 1:1.8 mixture of thiol-ending **18** (6.9 mg, 4.70 μmol) and αManC_5_SH **14** (2.4 mg, 8.37 μmol) in CD_3_OD (0.97 mL) was used to obtain 1.7 mg of AuGNP **5**. TEM (average diameter): 2.1 ± 0.5 nm. Quantitative ^1^H-NMR (400 MHz, D_2_O containing 0.05 wt.% of 3-(trimethylsilyl)propionic-*2,2,3,3-d*_4_ acid, sodium salt as an internal standard): 0.60 mg of **5** were dissolved in 180 μL of D_2_O and 40 μL of D_2_O containing 0.05 wt.% TSP were added and 151 nmoles of DNJ conjugate were found. Significant peaks: δ = 7.81 (br s, 3H, triazole from DNJ derivative), 2.57-2.44 (m, 6H, from DNJ conjugate) ppm. UV-Vis (H_2_O, 0.1 mg/mL): absence of a maximum band at around 520 nm.

### 3.2. Biological Evaluation 

*p*-Nitrophenyl-α-d-mannopyranoside and α-mannosidase (EC 3.2.1.24, from Jack Bean, *Km* = 2.0 mM pH 5.5) were purchased from Merck (Darmstadt, Germany). Inhibition constants were determined by spectrophotometrically measuring the residual hydrolytic activities of the mannosidase against *p*-nitrophenyl-α-d-mannopyranoside in the presence and absence of the inhibitor with a VersaMax Microplate Reader. All kinetics were performed at 25 °C and started by substrate addition (20 µL) in a 100 µL assay medium (acetate buffer, 0.2 M, pH = 5) containing α-mannosidase (0.015 U/mL), in presence or absence of various concentrations of inhibitor and substrate (concentrations from km/8 to 2 km). After 15–30 min incubation, the reaction was quenched by the addition of 1M Na_2_CO_3_ 100 µL). The absorbance of the resulting solution was determined at 405 nm. Under these conditions, the *p*-nitrophenolate released led to optical densities linear with both reaction time and concentration of the enzyme. *K_i_* values were determined, in duplicate or triplicate, using the Dixon or Lineweaver-Burk graphical methods with Microsoft Excel [37].

## 4. Conclusions

In conclusion, we report, in this work, the first example of gold nanoparticles decorated with the iminosugar deoxynojirimycin (DNJ). Jack bean α-mannosidase (JBα-man) was chosen as the target enzyme for the biological evaluation due to its well-known responsiveness to the multivalent presentation of iminosugar inhitopes. Monovalent and trivalent DNJ-derivative functionalized with a thiol were synthesized and used in mixture with a glucose or mannose thiol-ending inner component for the in-situ preparation of heterovalent AuGNPs. AuGNPs, bearing only the sugar component, were also prepared as control systems. Biological assays towards JBα-man revealed a competitive inhibition for the whole set of AuGNPs **1**–**5**, while glyco-coated AuGNPs **6**–**7** were inactive, thus demonstrating the fundamental role played by the iminosugar moiety for the inhibition. Comparison with the monovalent reference **19** highlighted affinity enhancements, per inhitope higher than 1600, for the AuGNPs **3**–**5** decorated with the trivalent DNJ-derivative, with the best result obtained for AuGNP **5** (*K_i_* = 84 nM, *rpn* = 3833). Interestingly, AuGNPs **2** and **5** share similar concentration in DNJ but vary by their local inhitope density, which makes them powerful tools to measure the impact of DNJ distribution onto the affinity enhancements. The significant gain of affinity, observed between the DNJ-coated and DNJ dendron-coated AuGNPs, highlights the importance of the bind-and-recapture effect in the complex, interconnected mechanisms underlying the inhibitory multivalent effects. Further investigation with these new nanosystems is currently ongoing in our laboratories. 

## Figures and Tables

**Table 1:** Inhibitory activity of AuGNPs **1**–**7** towards Jack bean α-mannosidase (JBα-man).

AuGNP	DNJ Conc. (μM) in 2 mg mL^−1^ AuGNP ^1^	*K_i_* (μM) ^2^	*rpn* ^3^
**1**	413	16 ± 2	20
**2**	467	8 ± 2	40
**3**	567	0.198 ± 0.060	1626
**4**	450	0.175 ± 0.171	1840
**5**	503	0.084 ± 0.066	3833
**6**	0	n.i. ^4^	^_^
**7**	0	n.i. ^4^	^_^

^1^ Estimated by qNMR. ^2^ The inhibition constant unit directly reflects the concentration of DNJ and not the concentration of nanoparticles. ^3^ Relative potency per DNJ unit, with respect to the monovalent reference **19**; *rpn* = *K_i_* (monovalent reference **19**)/*K_i_* (DNJ in AuGNP **1**–**5**). ^4^ n.i. = no inhibition at 0.5 mg mL^−1^.

## Supplementary Materials

The following are available online. The ^1^H and ^13^C-NMR spectra of DNJ-based ligands **9**, **11**, **16** and **17**; the characterization of AuGNPs **1**–**5** (^1^H-NMR spectra, TEM graphs, UV-Vis spectra); the Dixon and Lineweaver-Burk plots for determination of inhibition constants of AuGNPs **1**–**5**; Table S1 which contains a resume of DNJ-based AuGNPs 1–5 characterization (average size diameter, calculated amount of iminosugar).

## Sample Availability

Samples of the compounds reported in the manuscript are not available from the authors.
