# Supplementary material for: Hybrid Multivalent Jack Bean α-Mannosidase Inhibitors: The First Example of Gold Nanoparticles Decorated with Deoxynojirimycin Inhitopes
Source: Molecules. 2021 Sep 27;26(19):5864. doi: 10.3390/molecules26195864 (PMC8512634; doi:10.3390/molecules26195864)
Supplement: Supplementary file 1 [file molecules-26-05864-s001.zip › molecules-1386862-supplementary.pdf]

Hybrid Multivalent Jack Bean α-Mannosidase Inhibitors: The First Example of Gold Nanoparticles Decorated with Deoxynojirimycin Inhitopes

Costanza Vanni 1, Anne Bodlenner 2,*, Marco Marradi 1, Jérémy P. Schneider 2, Maria de los Angeles Ramirez 3, Sergio Moya 3, Andrea Goti 1,4, Francesca Cardona 1,4, Philippe Compain 2 and Camilla Matassini 1,4,*

1 Dipartimento di Chimica "Ugo Schiff", Università di Firenze, Via della Lastruccia 3-13, 50019 Sesto Fiorentino, Italy; costanza.vanni@unifi.it (C.V.); marco.marradi@unifi.it (M.M.); andrea.goti@unifi.it (A.G.); francesca.cardona@unifi.it (F.C.)

2 Laboratoire d'Innovation Moléculaire et Applications (LIMA), University of Strasbourg, University of Haute-Alsace, CNRS (UMR 7042), Equipe de Synthèse Organique et Molécules Bioactives (SYBIO), ECPM, 25 Rue Becquerel, 67000 Strasbourg, France; jeremy.schneider@live.fr (J.P.S.); philippe.compain@unistra.fr (P.C.)

3 Soft Matter Nanotechnology Lab, CIC biomaGUNE, Basque Research and Technology Alliance (BRTA), Paseo Miramón 182, 20014 Donostia-San Sebastián, Gipuzkoa, Spain; aangie.ramirez@gmail.com (M.d.l.A.R.); smoya@cicbiomagune.es (S.M.)

4 Associated with LENS, Via N. Carrara 1, 50019 Sesto Fiorentino, Italy

* Correspondence: annebod@unistra.fr (A.B.); camilla.matassini@unifi.it (C.M.); Tel.: +33-36-8852684 (A.B.); +39-055-457-3536 (C.M.)

Supplementary Materials

Table of contents

^{1}H-NMR and ^{13}C-NMR spectra of compound **9** (Figures S1-S3)	S3
^{1}H-NMR and ^{13}C-NMR spectra of compound **11** (Figures S4-S6)	S5
^{1}H-NMR and ^{13}C-NMR spectra of compound **16** (Figures S7-S9)	S7
^{1}H-NMR and ^{13}C-NMR spectra of compound **17** (Figures S10-S12)	S9
Characterization of AuGNP **1** (Figures S13-S16)	S11
Characterization of AuGNP **2** (Figures S17-S20)	S13
Characterization of AuGNP **3** (Figures S21-S24)	S15
Characterization of AuGNP **4** (Figures S25-S28)	S17
Characterization of AuGNP **5** (Figures S29-S32)	S19
Characterization Table for AuGNP **1-7** (Table S1)	S21
Inhibition assays of AuGNPs **1**-**5** (Figures S33-S37)	S23

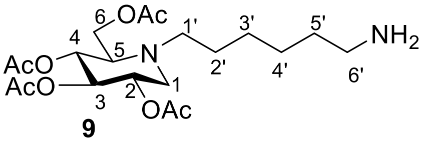

9

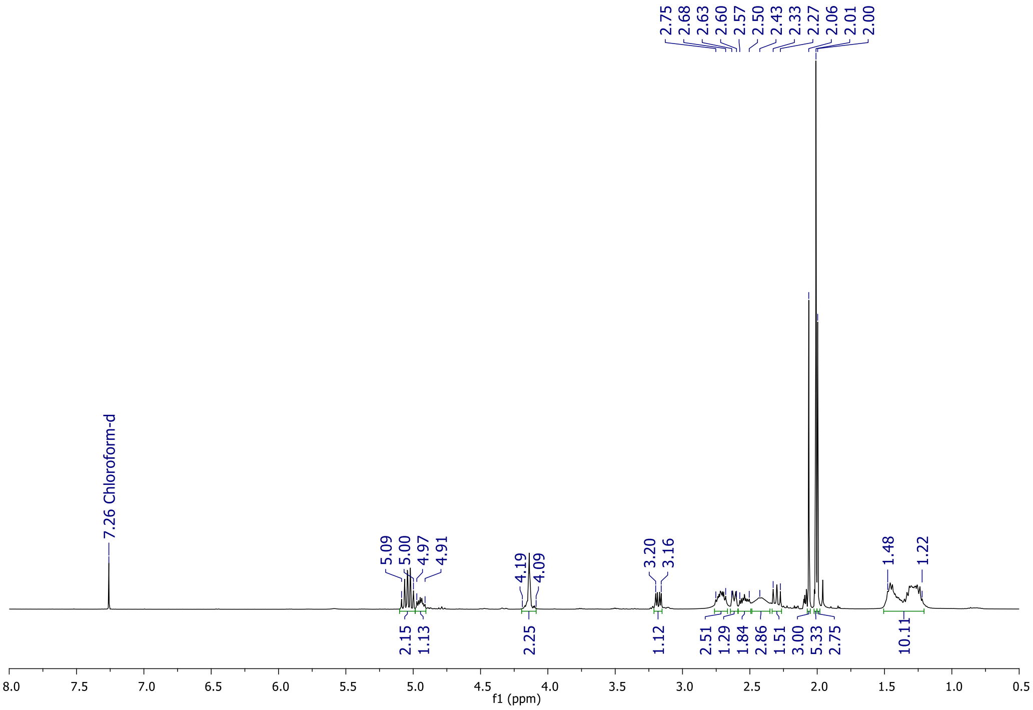

Figure S1. ^{1}H-NMR spectrum of compound **9** (400 MHz, $CDCl_3$).

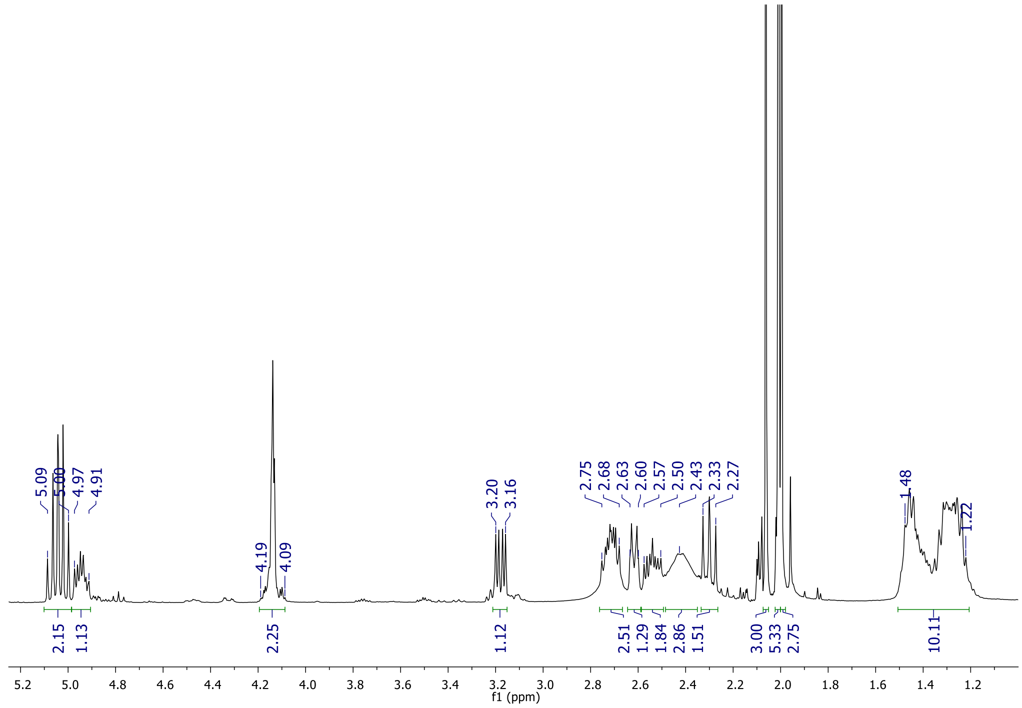

Figure S2. Expansion of ^{1}H-NMR spectrum of compound **9** (400 MHz, $CDCl_3$).

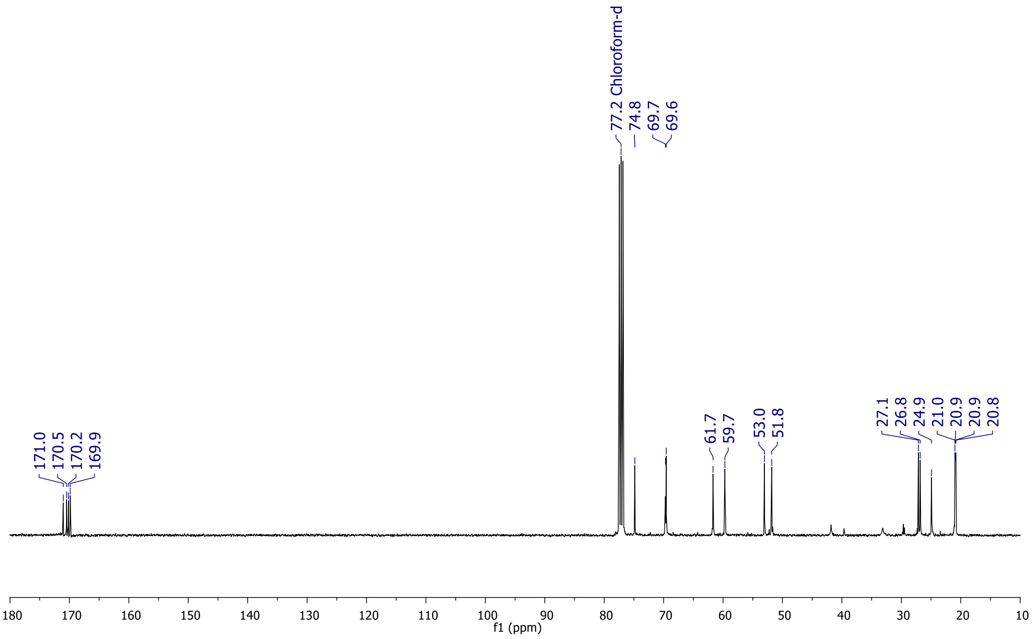

Figure S3. ^{13}C-NMR spectrum of compound **9** (100 MHz, $CDCl_3$).

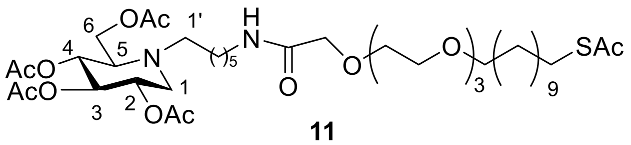

11

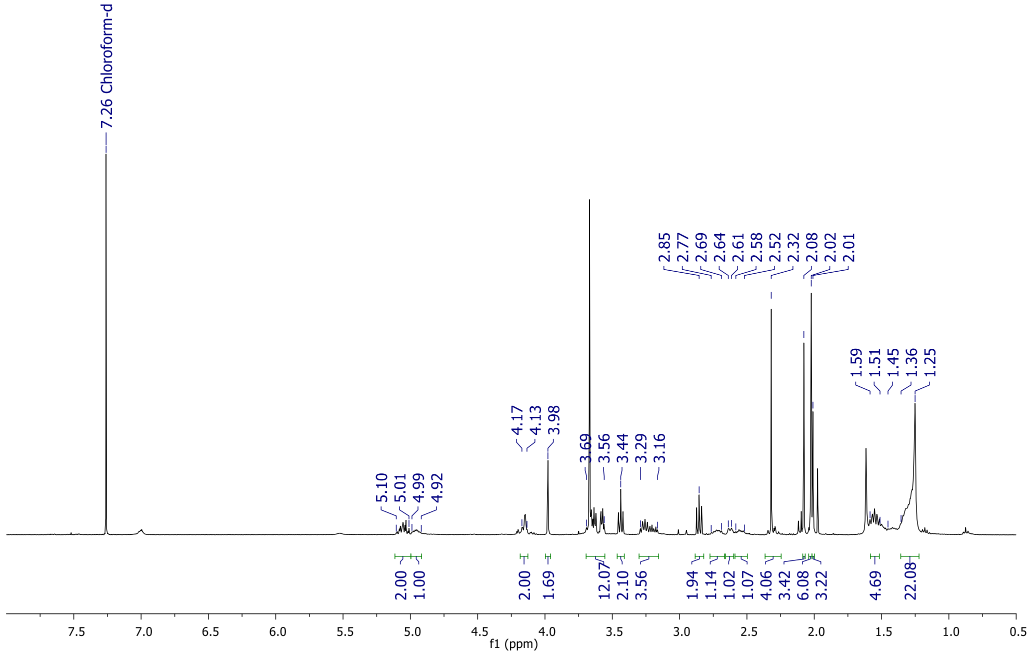

Figure S4. ^{1}H-NMR spectrum of compound **11** (400 MHz, $CDCl_3$).

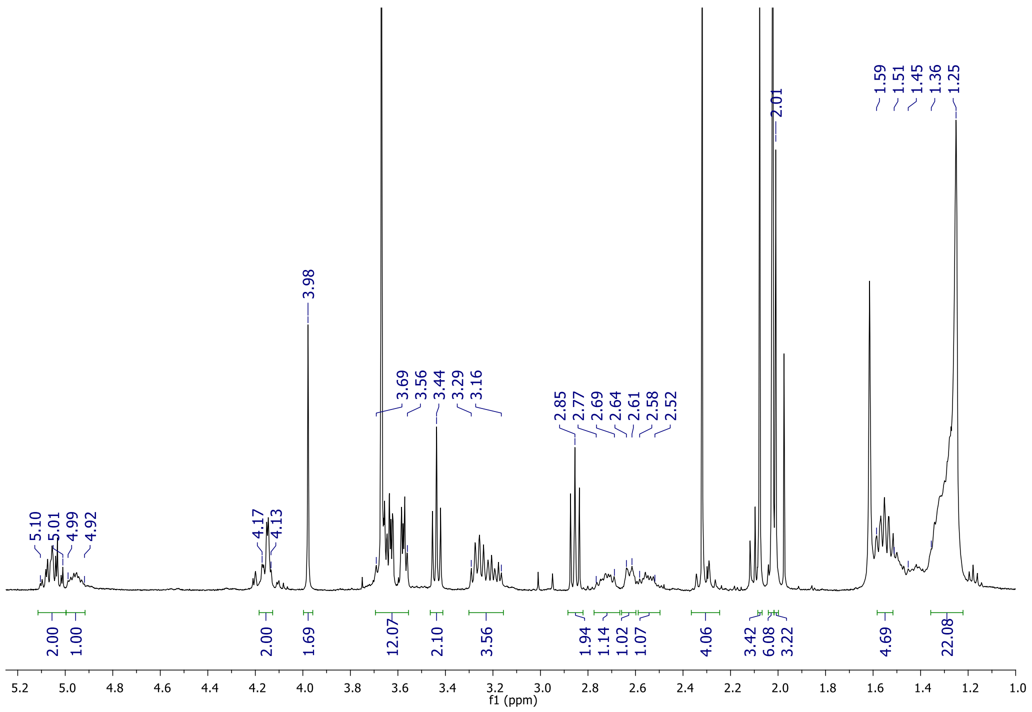

Figure S5. Expansion of ^{1}H-NMR spectrum of compound **11** (400 MHz, $CDCl_3$).

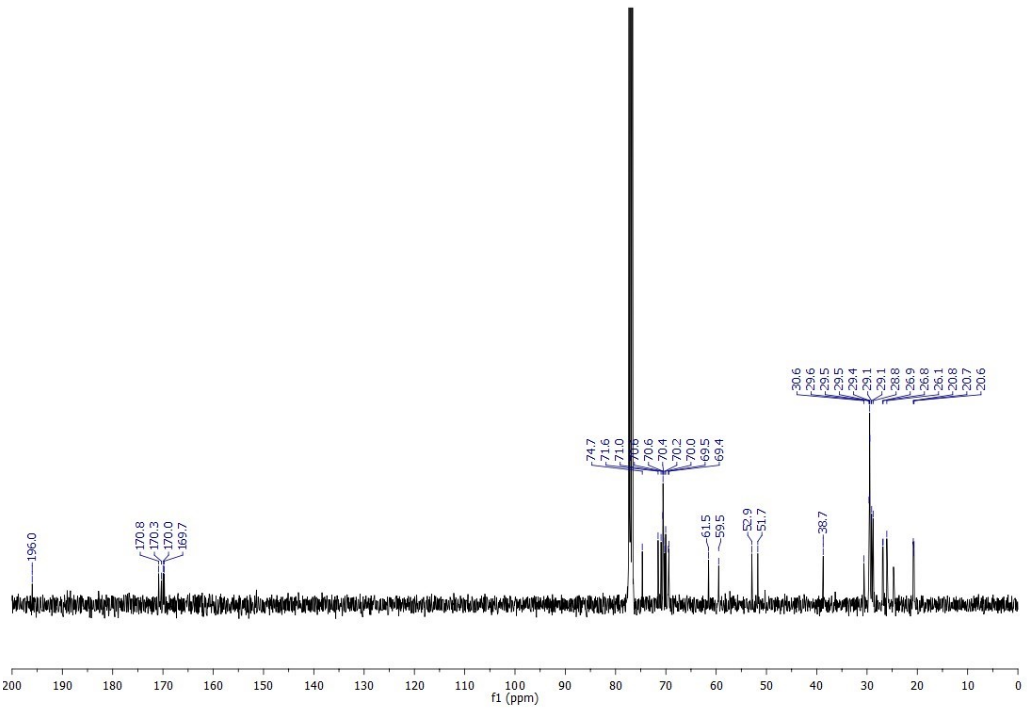

Figure S6. ^{13}C-NMR spectrum of compound **11** (100 MHz, $CDCl_3$).

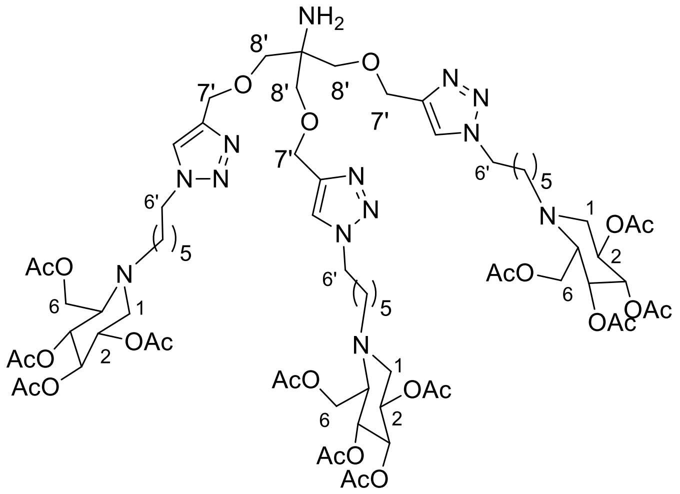

16

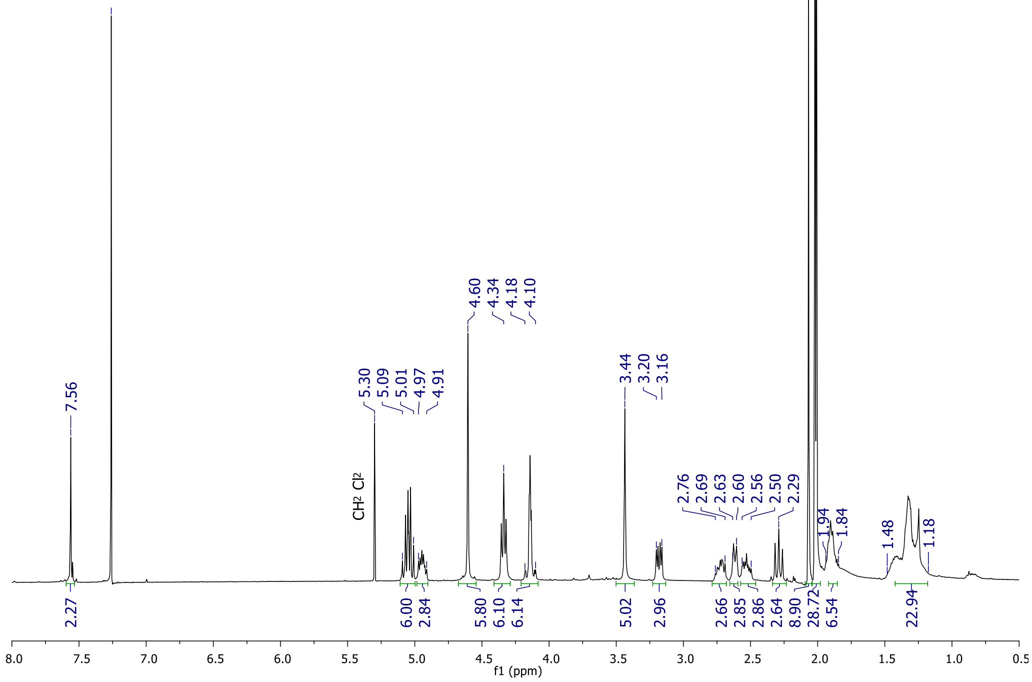

Figure S7. ^{1}H-NMR spectrum of compound **16** (400 MHz, $CDCl_3$).

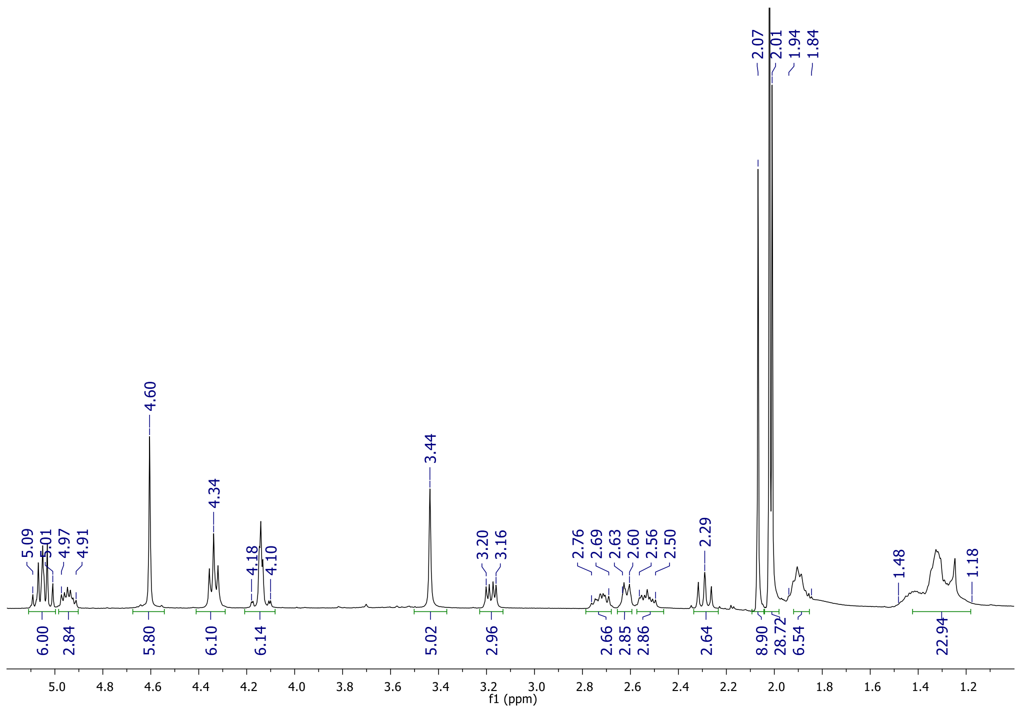

Figure S8. Expansion of ^{1}H-NMR spectrum of compound **16** (400 MHz, $CDCl_3$).

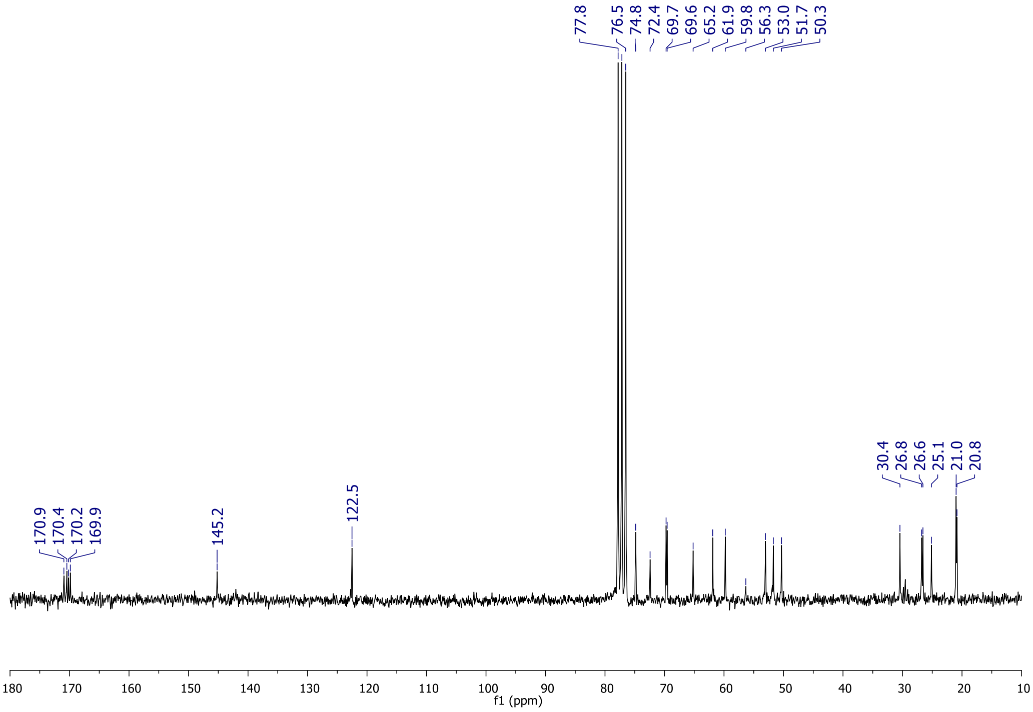

Figure S9. ^{13}C-NMR spectrum of compound **16** (100 MHz, $CDCl_3$).

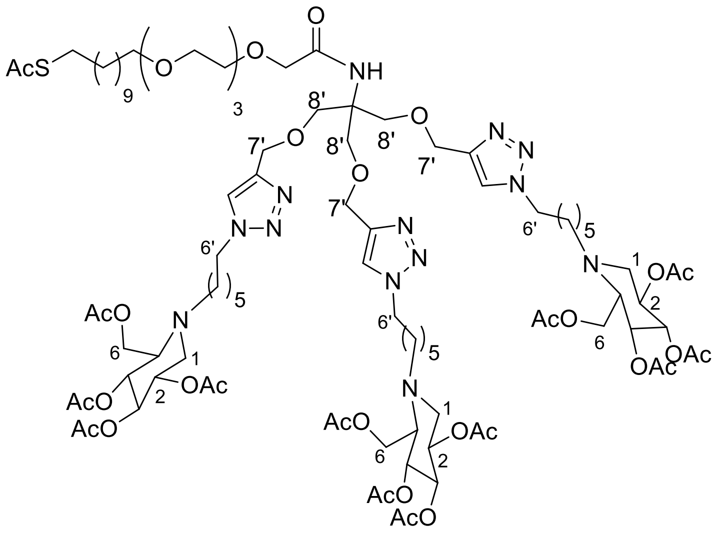

17

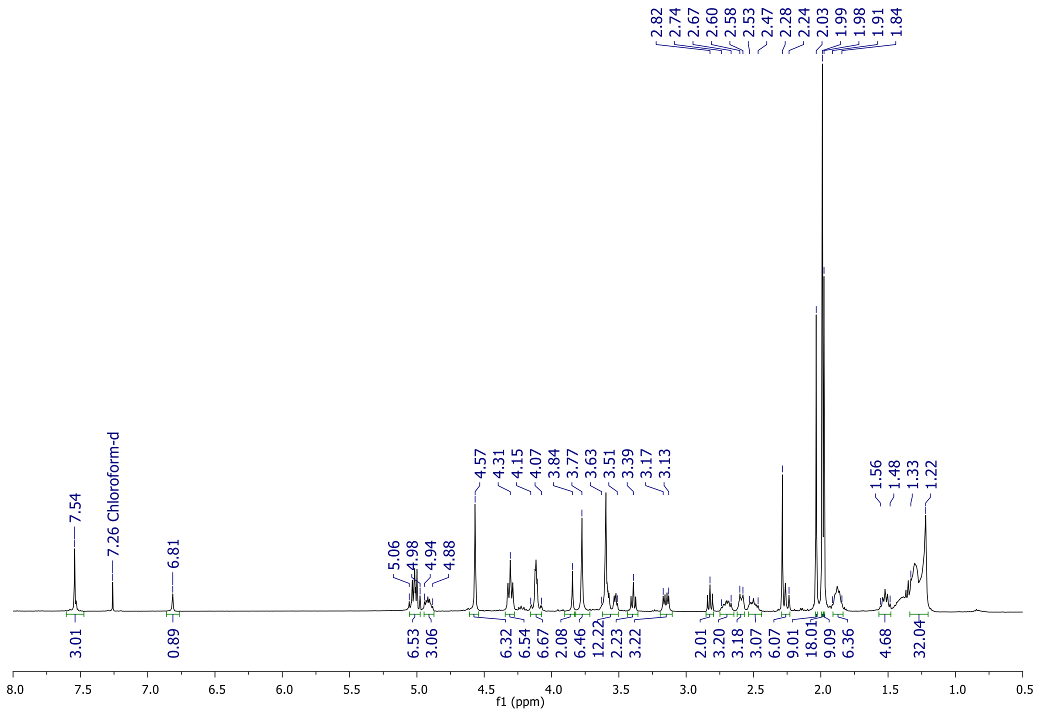

Figure S10. ^{1}H-NMR spectrum of compound **17** (400 MHz, $CDCl_3$).

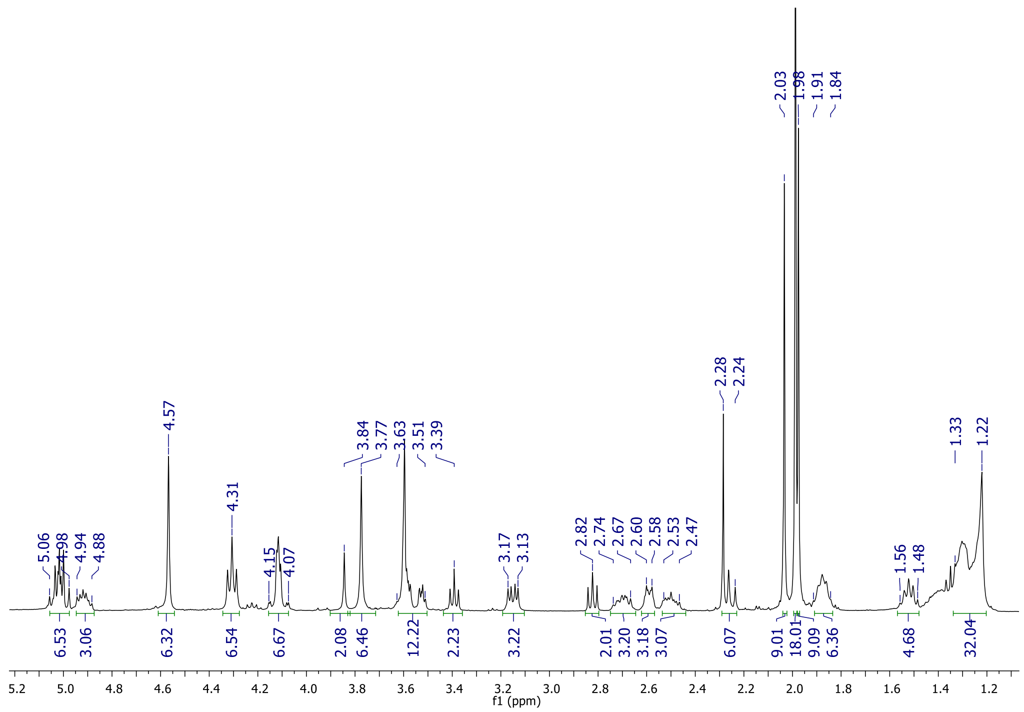

Figure S11. Expansion of ^{1}H-NMR spectrum of compound **17** (400 MHz, $CDCl_3$).

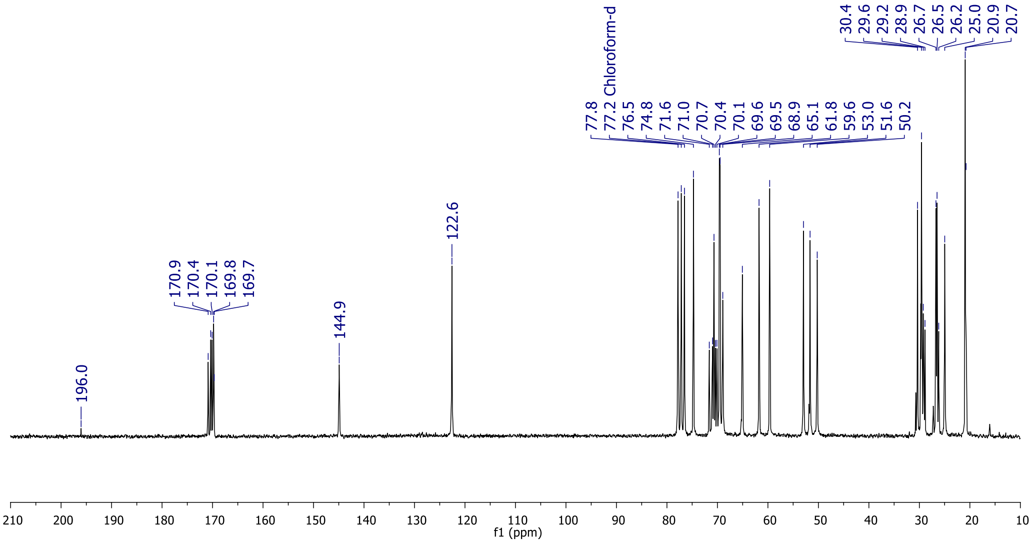

Figure S12. ^{13}C-NMR spectrum of compound **17** (100 MHz, $CDCl_3$).

*Characterization of AuGNP **1***

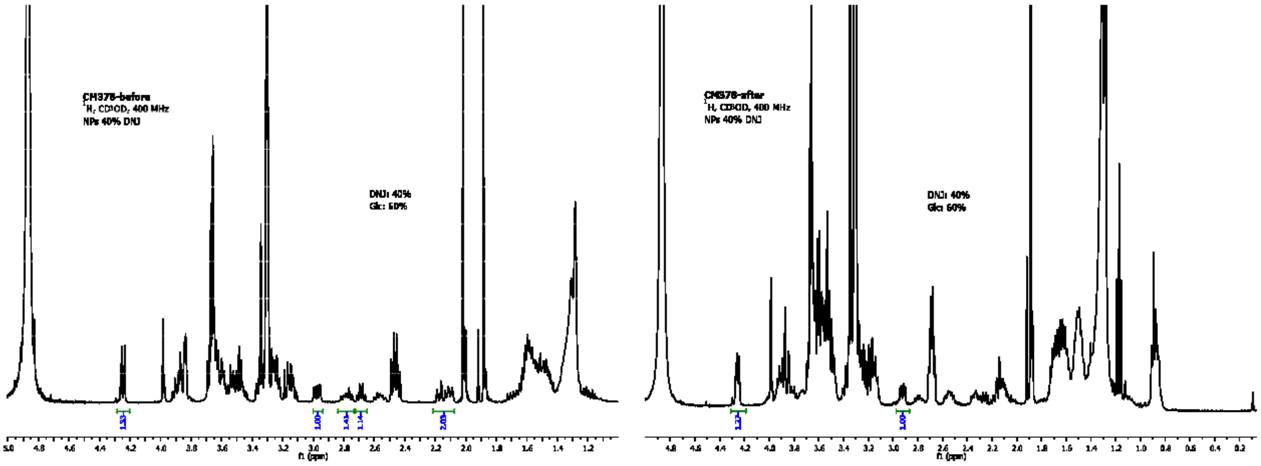

Figure S13. ^{1}H NMR of sugar/iminosugar ligands mixture before (left) and after (right) formation of AuGNP **1** (400 MHz, CD_3OD).

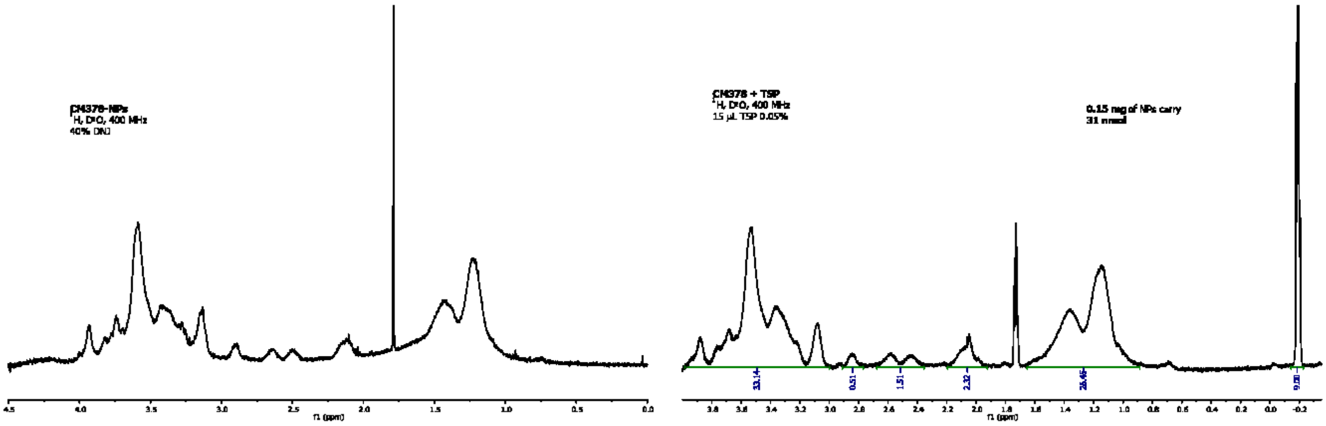

Figure S14. ^{1}H NMR and ^{1}H qNMR with TSP-d_4 of AuGNP **1** (400 MHz, D_2O).

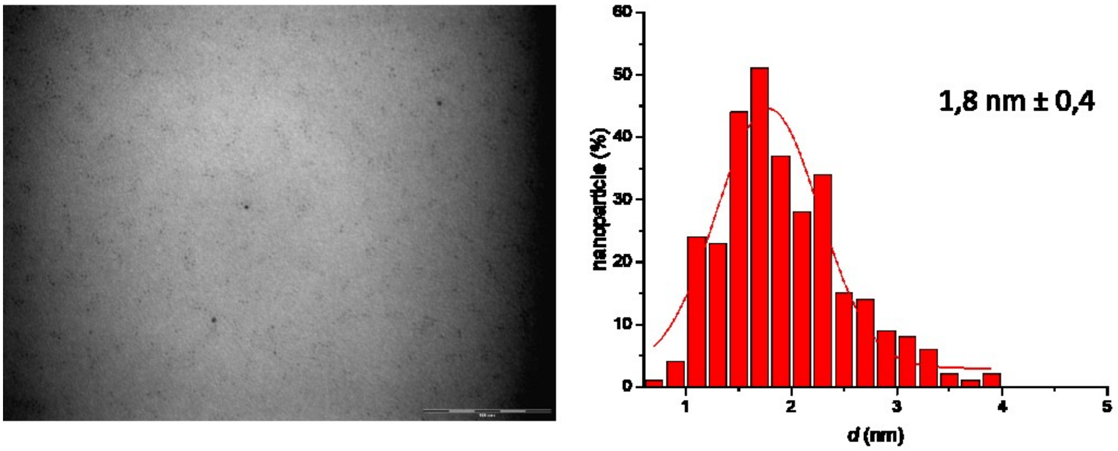

Figure S15. TEM micrograph (scale = 100 nm) in H_2O and size-distribution histogram obtained by measuring 300 nanoparticles of AuGNP **1** (average diameter: 1.8 ±0.4 nm).

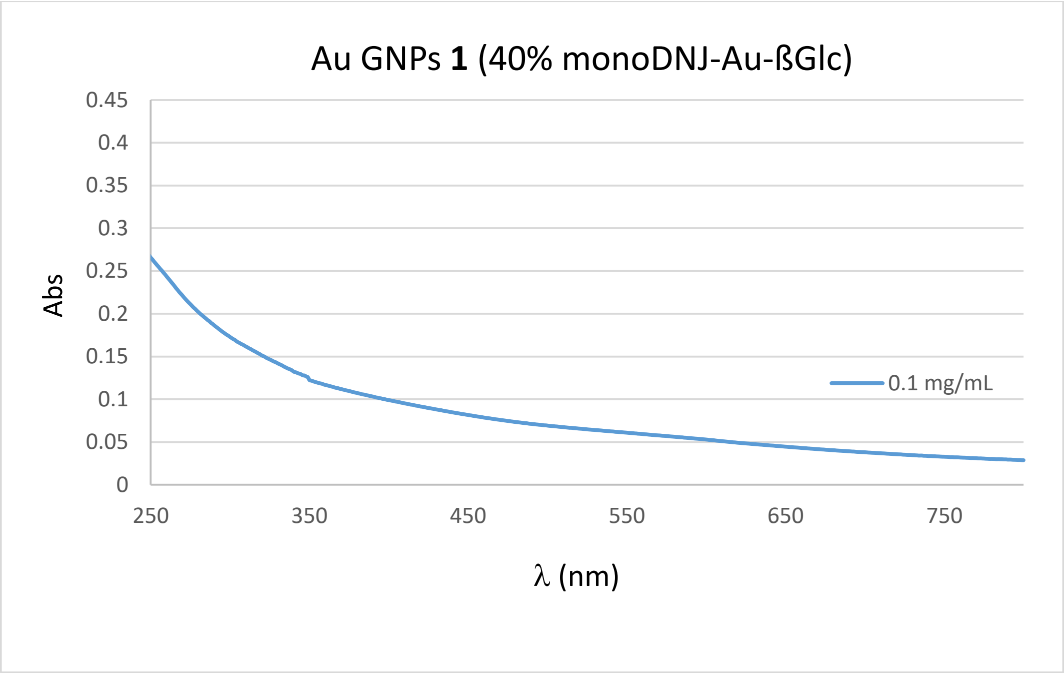

Figure S16. UV/vis spectrum of H_2O solution of AuGNP **1** recorded at concentration of 0.1 mg/mL.

*Characterization of AuGNP **2***

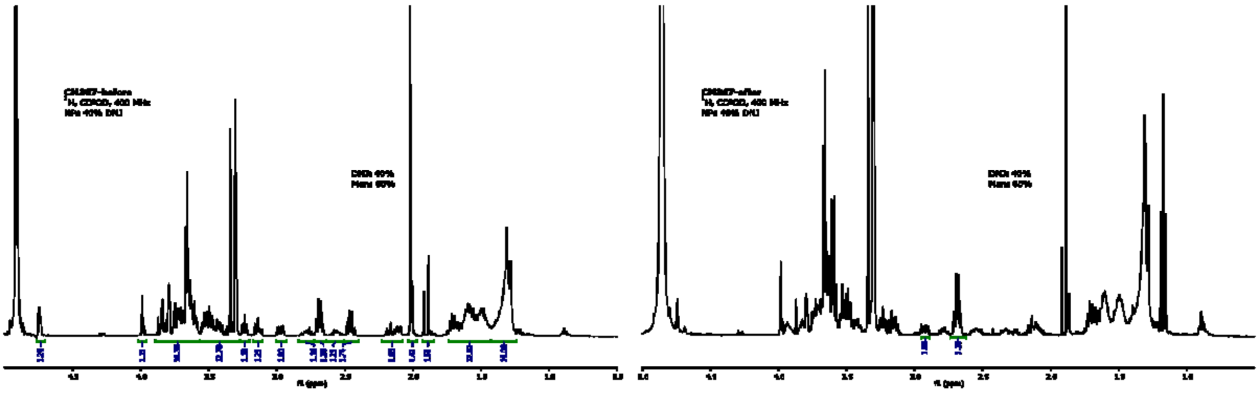

Figure S17. ^{1}H NMR of sugar/iminosugar ligands mixture before (left) and after (right) formation of AuGNP **2** (400 MHz, CD_3OD).

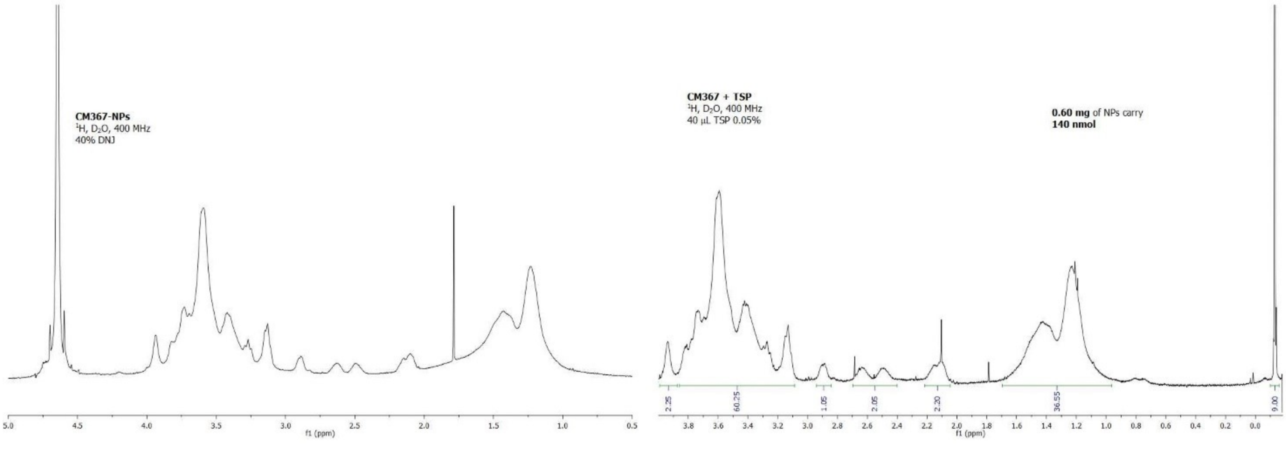

Figure S18. ^{1}H NMR and ^{1}H qNMR with TSP-d_4 of AuGNP **2** (400 MHz, D_2O).

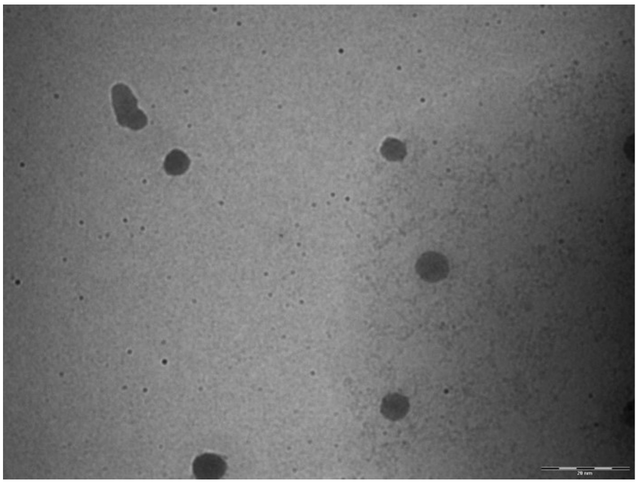

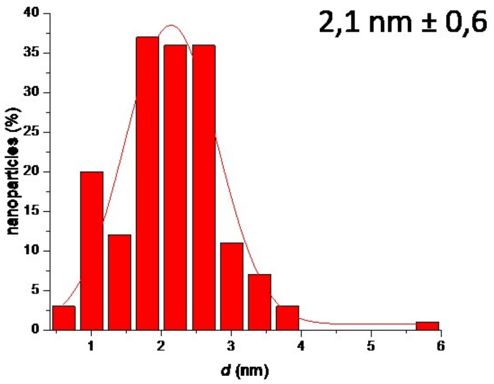

Figure S19. TEM micrograph (scale = 20 nm) in H_2O and size-distribution histogram obtained by measuring 300 nanoparticles of AuGNP **2** (average diameter: 2.1 ±0.6 nm, less than 3% shows a >5 nm diameter).

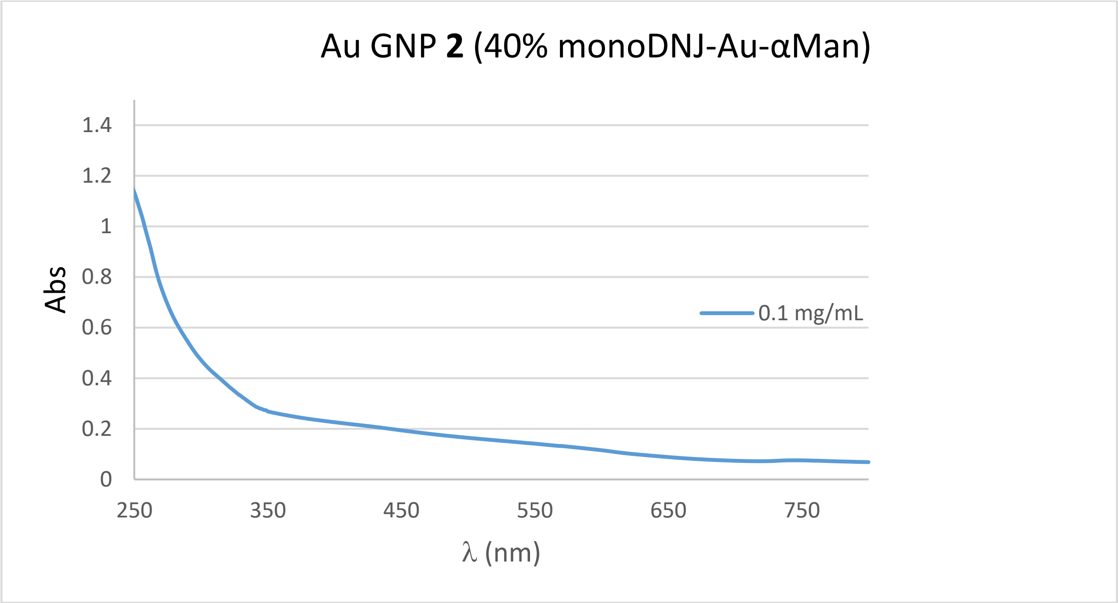

Figure S20. UV/vis spectrum of H_2O solution of AuGNP **2** recorded at concentration of 0.1 mg/mL.

Characterization of AuGNP ***3***

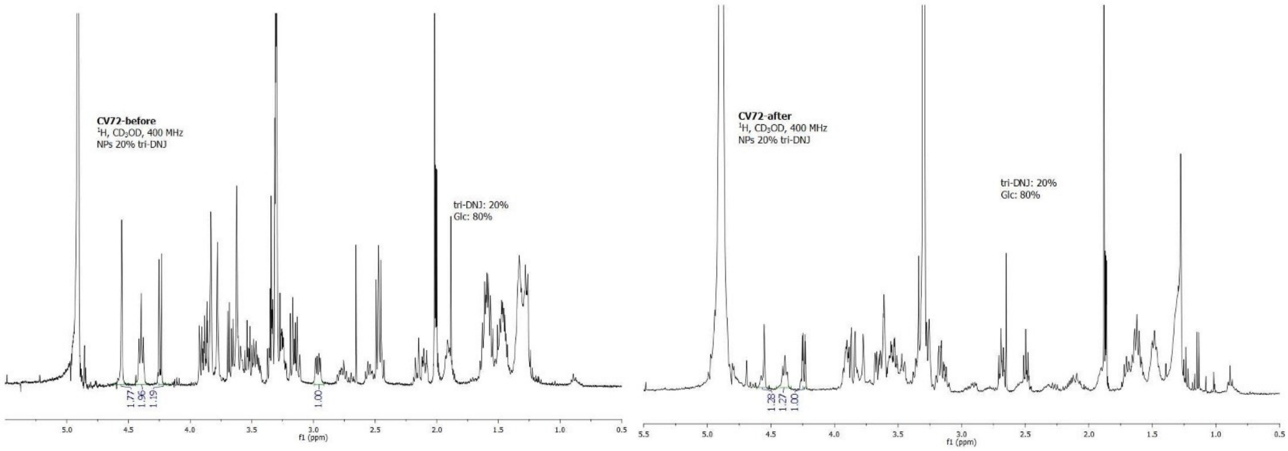

Figure S21. ^{1}H NMR of sugar/iminosugar ligands mixture before (left) and after (right) formation of AuGNP **3** (400 MHz, CD_3OD).

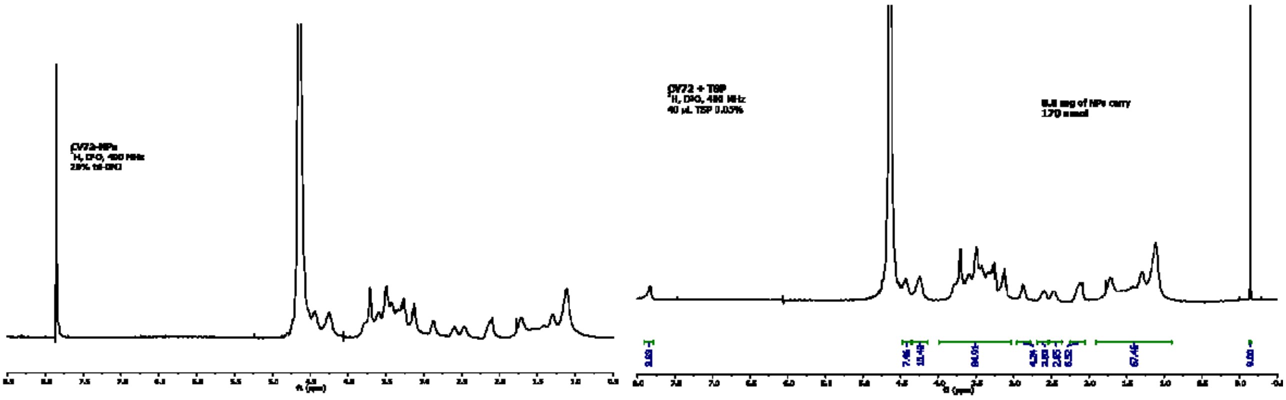

Figure S22. ^{1}H NMR and ^{1}H qNMR with TSP-d_4 of AuGNP **3** (400 MHz, D_2O).

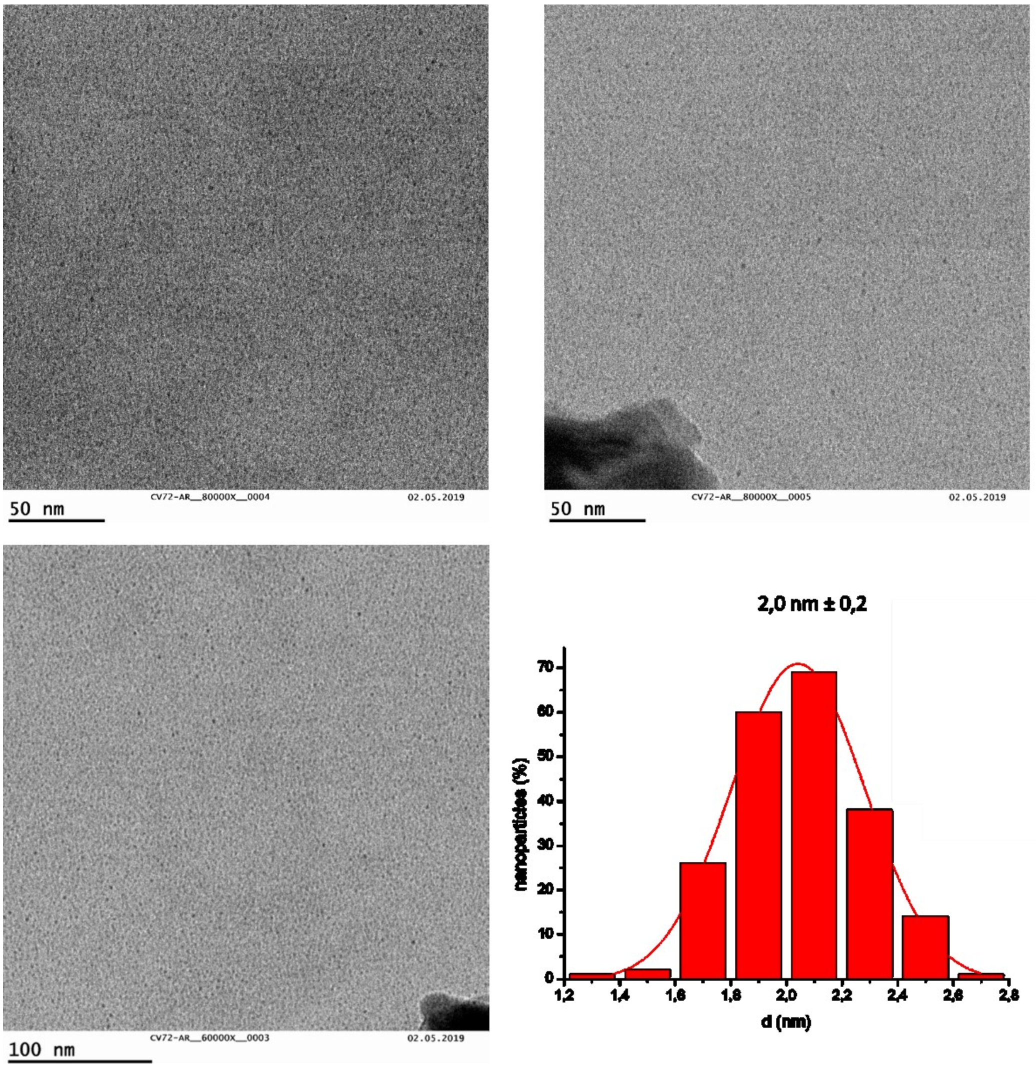

Figure S23. TEM micrographs in H_2O (magnification 80000x and 60000x) and size-distribution histogram obtained by measuring 300 nanoparticles of AuGNP **3** (average diameter: 2.1 ±0.5 nm).

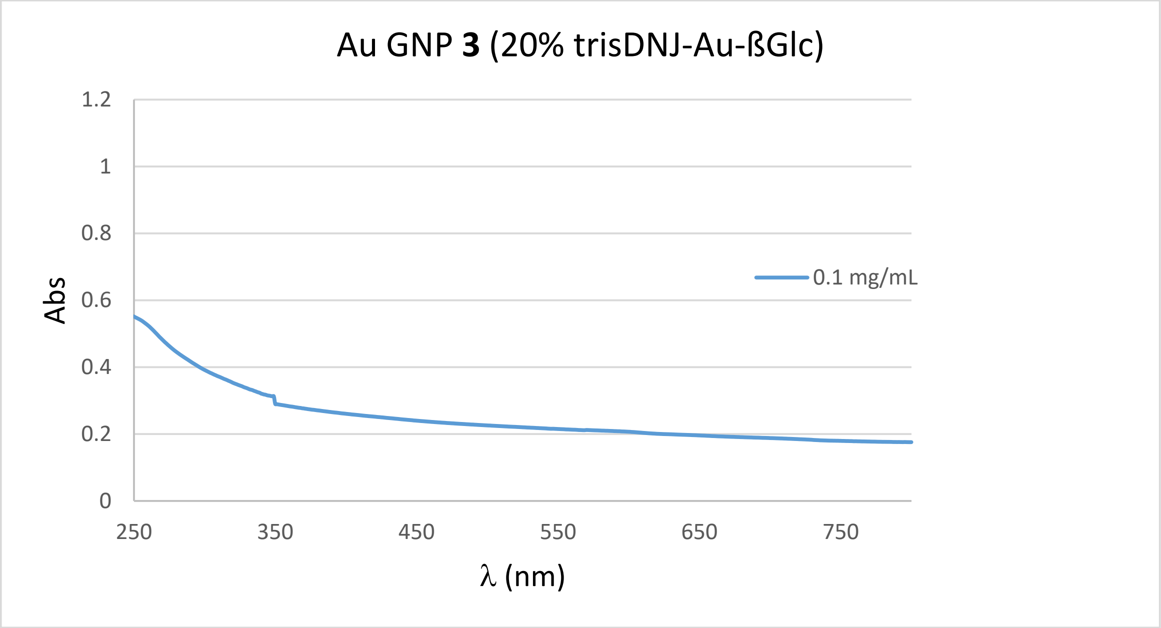

Figure S24. UV/vis spectrum of H_2O solution of AuGNP **3** recorded at concentration of 0.1 mg/ mL.

Characterization of AuGNP ***4***

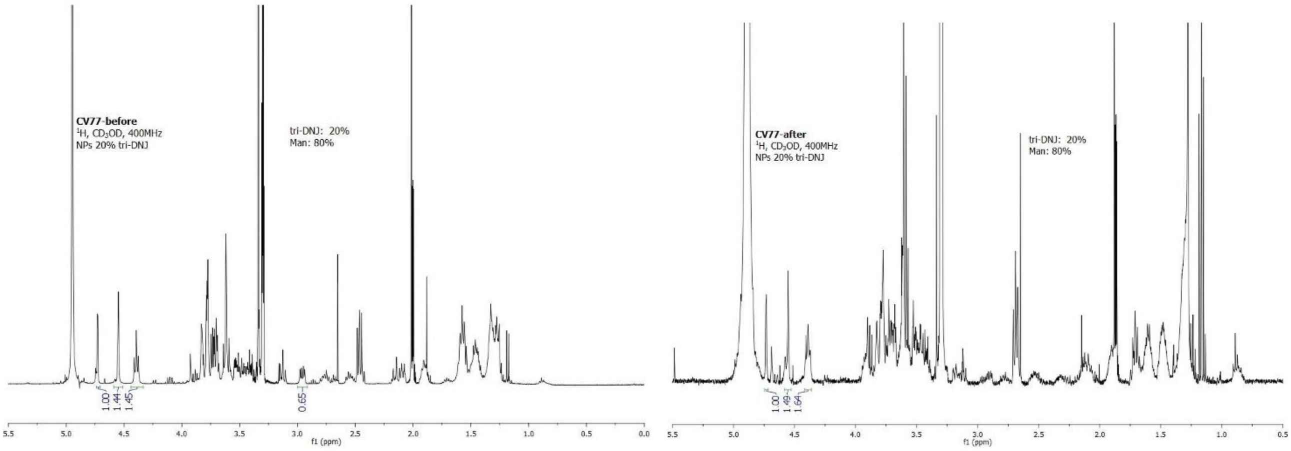

Figure S25. ^{1}H NMR of sugar/iminosugar ligands mixture before (left) and after (right) formation of AuGNP **4** (400 MHz, CD_3OD).

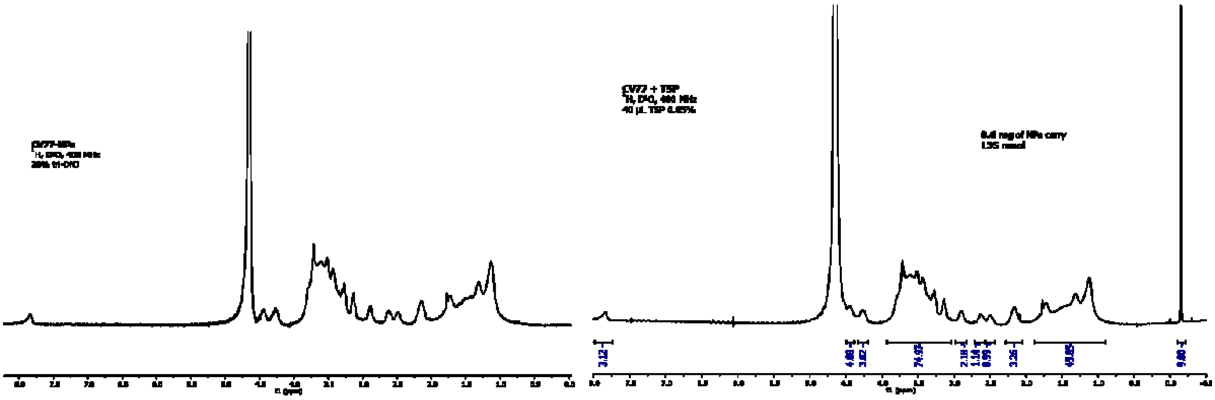

Figure S26. ^{1}H NMR and ^{1}H qNMR with TSP-d_4 of AuGNP **4** (400 MHz, D_2O).

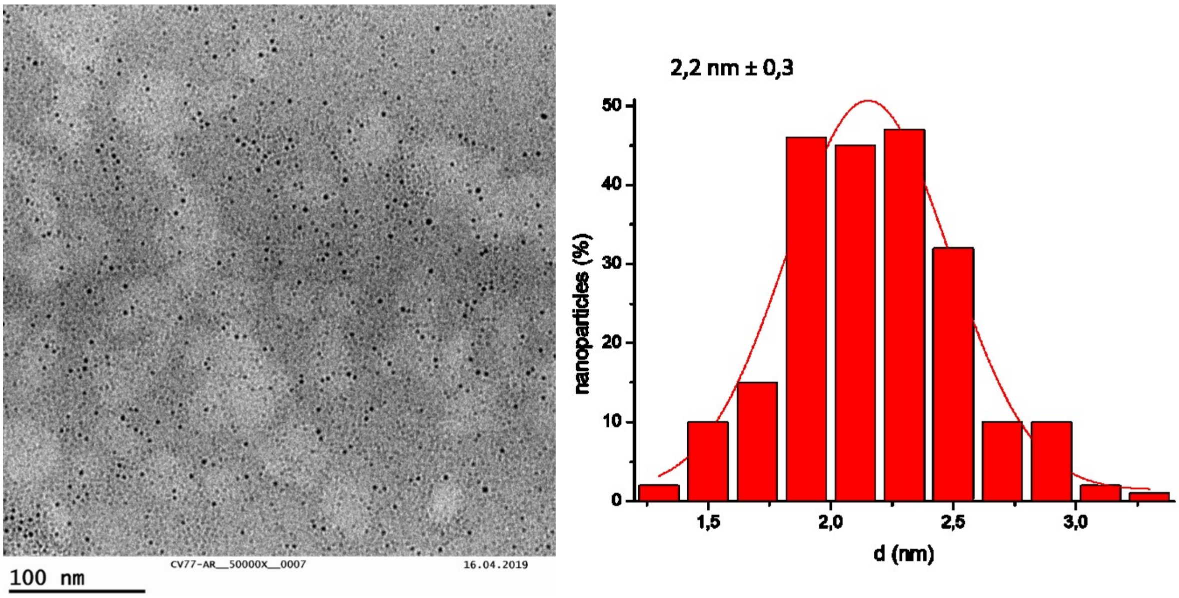

Figure S27. TEM micrograph in H_2O and size-distribution histogram obtained by measuring 300 nanoparticles of AuGNP **4** (average diameter: 2.0 ±0.4 nm).

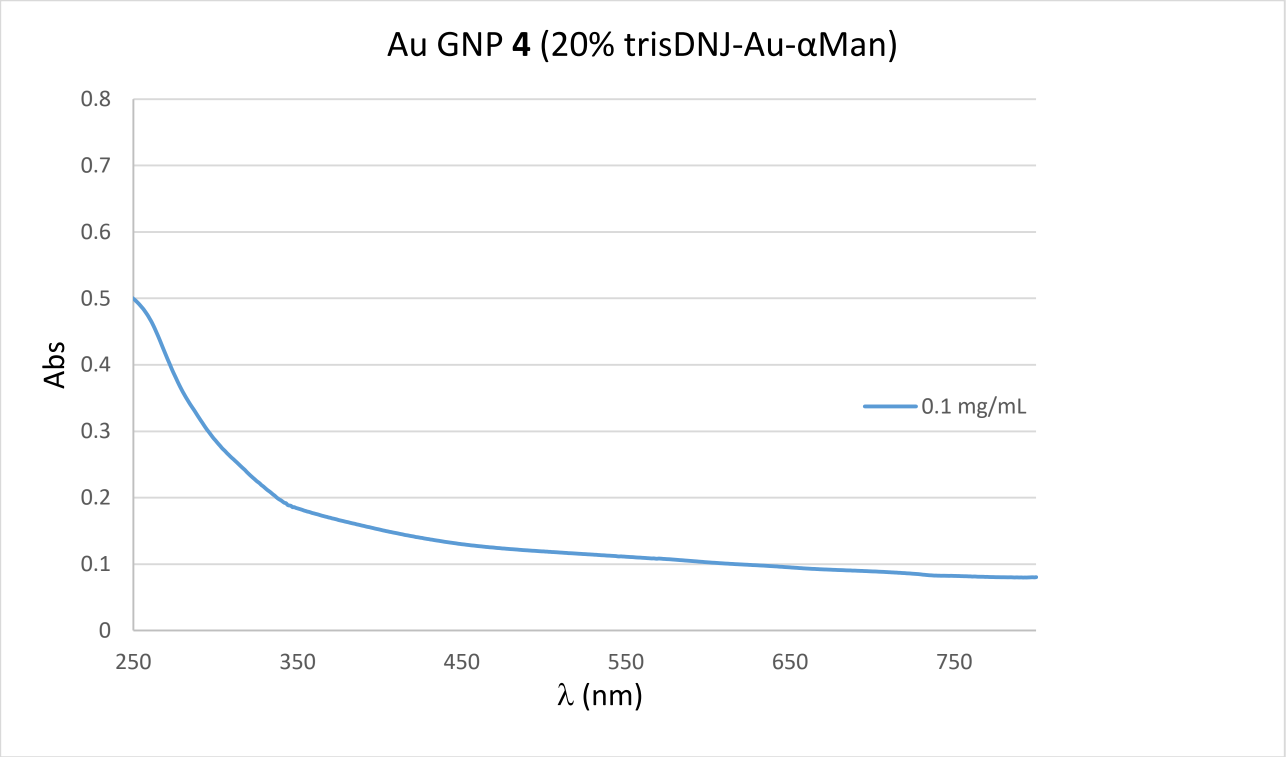

Figure S28. UV/vis spectrum of H_2O solution of AuGNP **4** recorded at concentration of 0.1 mg/mL.

*Characterization of AuGNP **5***

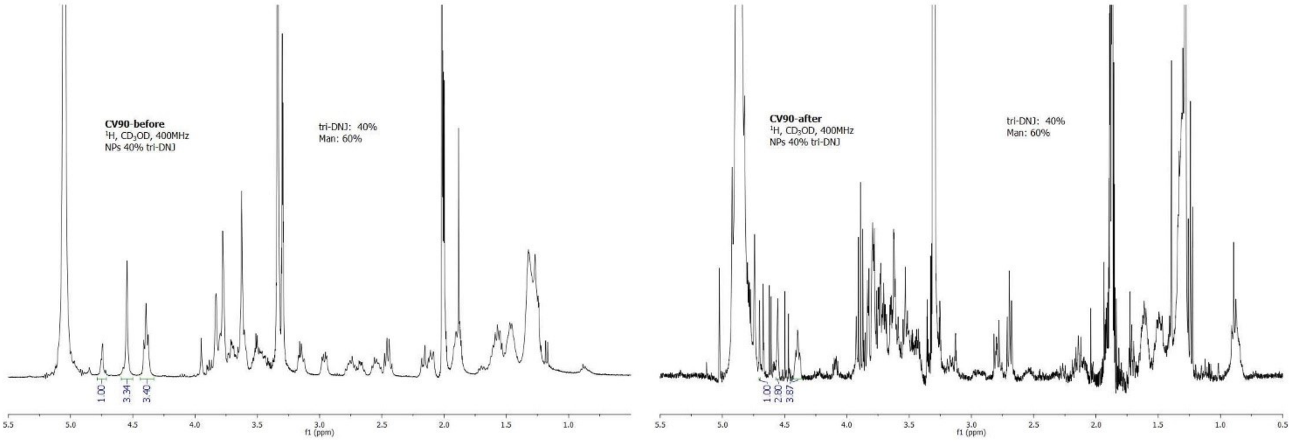

Figure S29. ^{1}H NMR of sugar/iminosugar ligands mixture before (left) and after (right) formation of AuGNP **5** (400 MHz, CD_3OD).

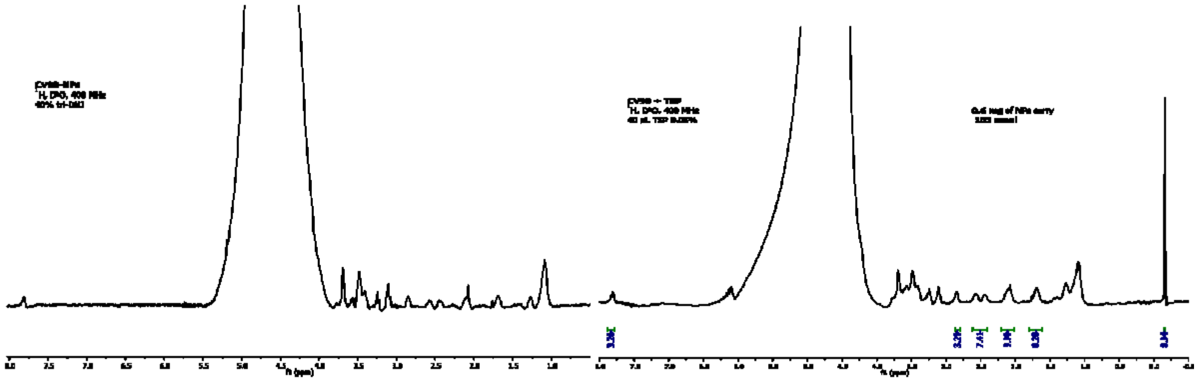

Figure S30. ^{1}H NMR and ^{1}H qNMR with TSP-d_4 of AuGNP **5** (400 MHz, D_2O).

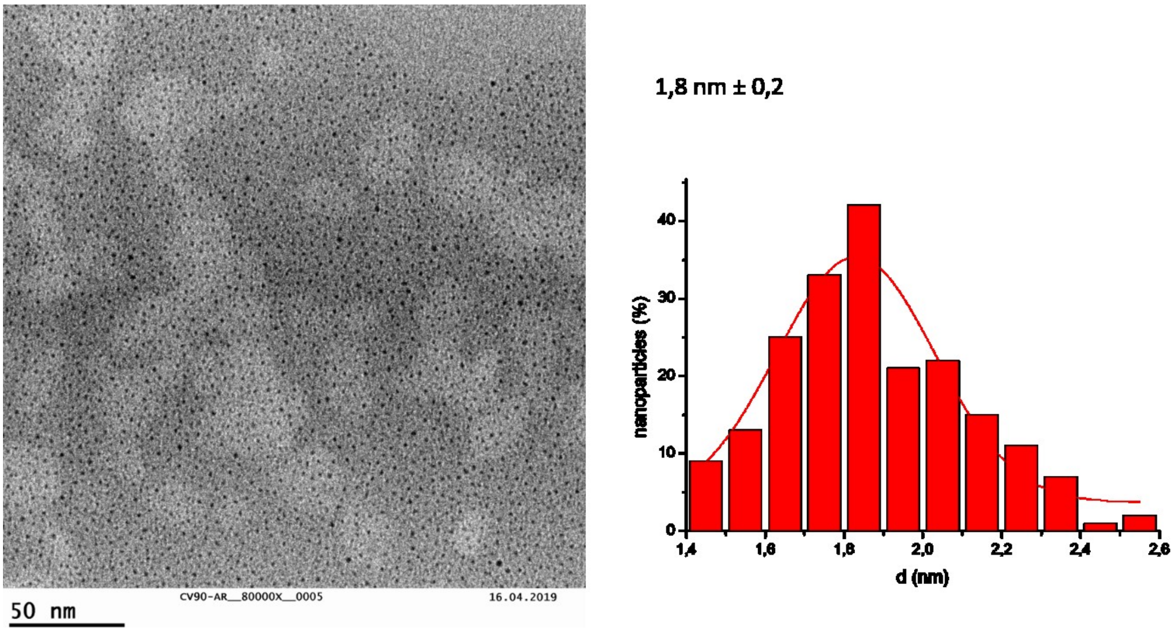

Figure S31. TEM micrograph in H_2O and size-distribution histogram obtained by measuring 300 nanoparticles of AuGNP **5** (average diameter: 2.1 ±0.5 nm).

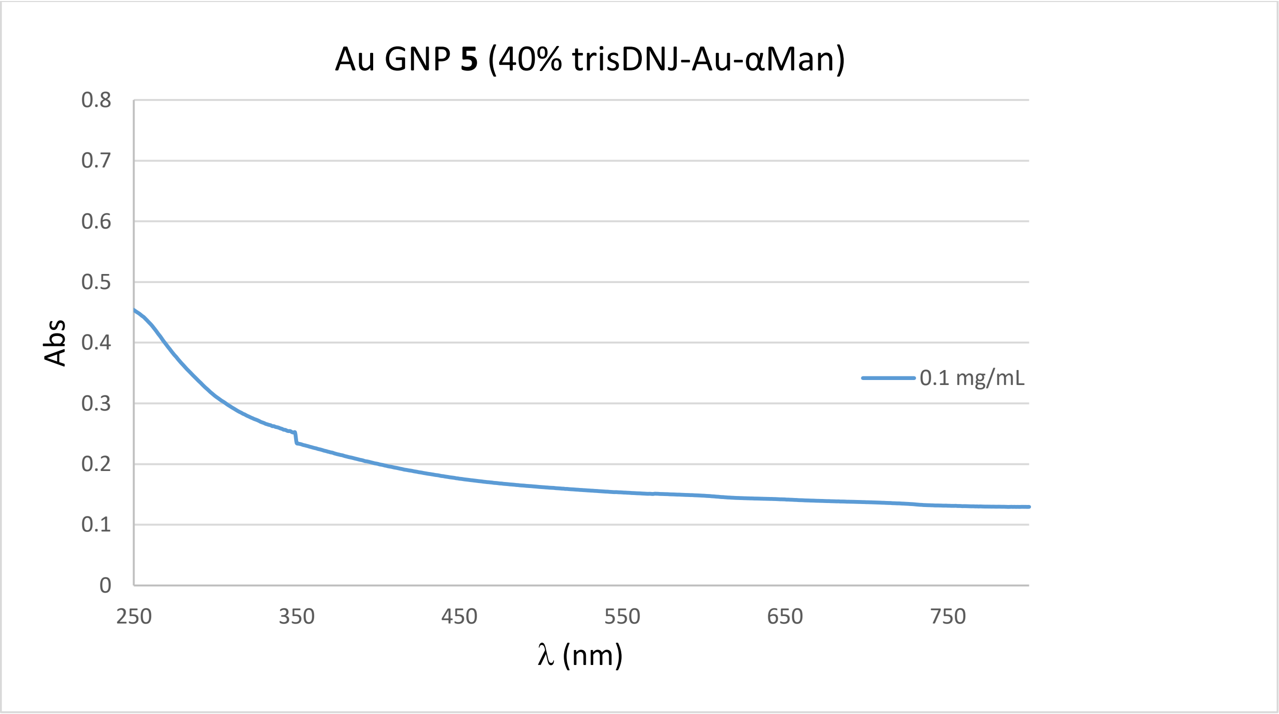

Figure S32. UV/vis spectrum of H_2O solution of AuGNP **5** recorded at concentration of 0.1 mg/mL.

Table S1. Summary table of DNJ-based AuGNPs **1**-**7** and their characterization.

Au-GNPs[1]	Characterization	
	Gold core size[2]	DNJ concentration for 2 mg/mL concentration of AuGNP[3]
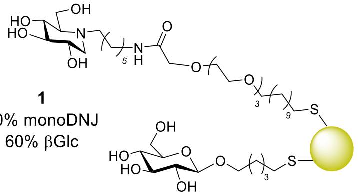 **1** 40% monoDNJ 60% βGlc	1.8±0.4 nm	413 μM
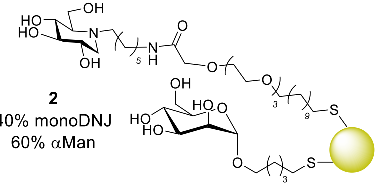 **2** 40% monoDNJ 60% αMan	2.1±0.6 nm	467 μM
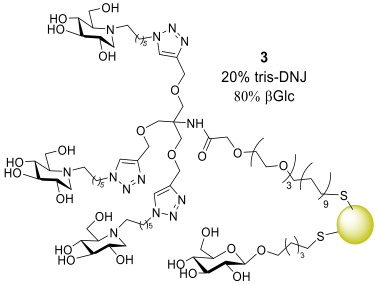 **3** 20% tris-DNJ 80% βGlc	2.1±0.5 nm	567 μM
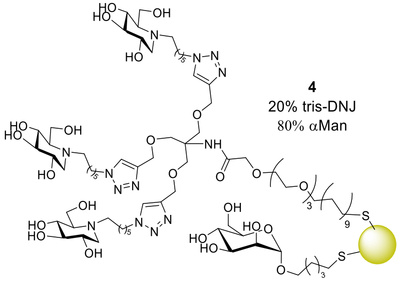 **4** 20% tris-DNJ 80% αMan	2.0±0.4 nm	450 μM

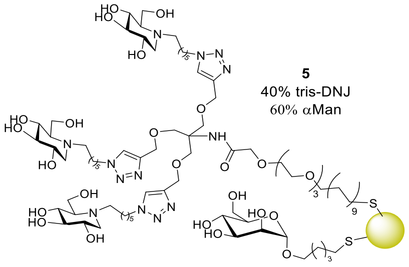 **5** 40% tris-DNJ 60% αMan	2.1±0.5 nm	503 μM
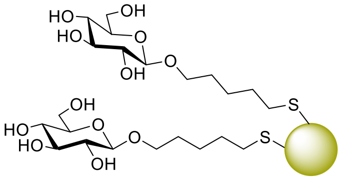 **6** 100% Au-βGlc	1.7±0.4 nm	/
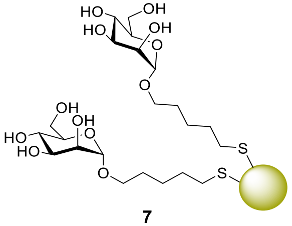 **7** 100% Au-αMan	1.6±0.4 nm	/

[1] The given percentages refer to the proportion of the ligands on the gold surface as determined by recording ^{1}H NMR spectrum of their initial mixture before the formation of the AuGNPs and confirmed by ^{1}H NMR spectrum of the supernatant after the AuGNPs formation. [2] Determined by Transmission Electron Microscopy (TEM). [3] Determined on the basis of qNMR analysis.

Inhibition assays

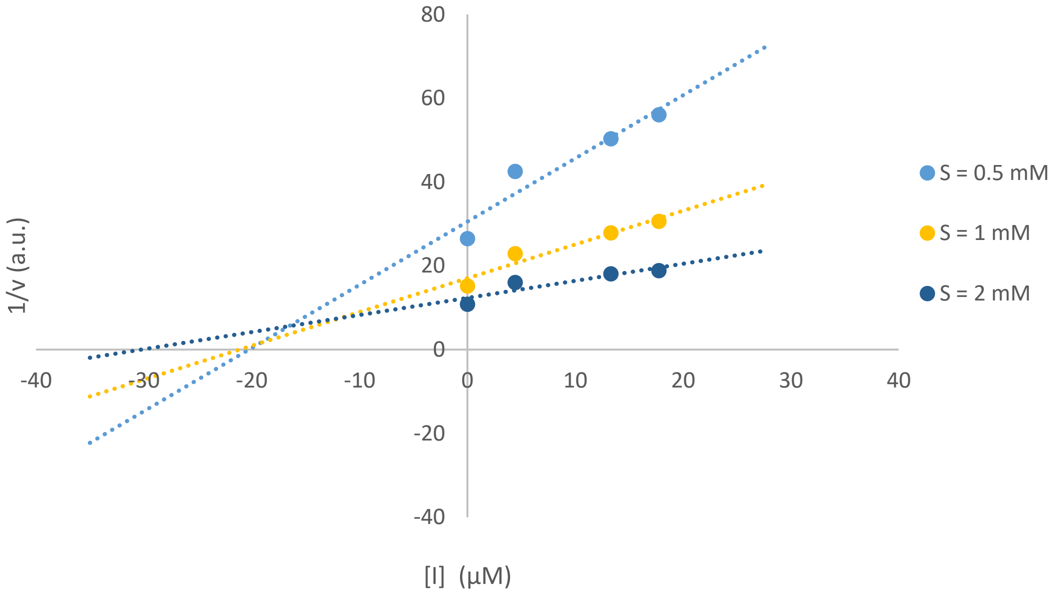

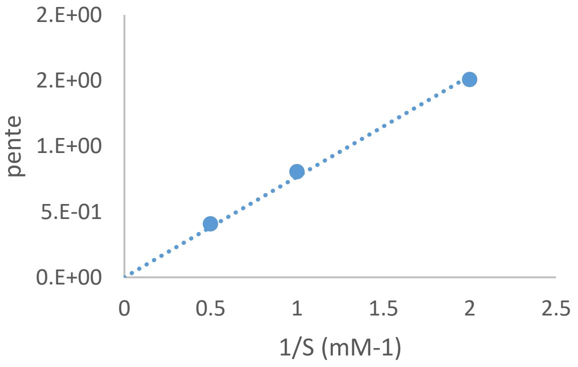

Figure S33. Dixon plot for K_i determination of compound AuGNP **1** against JBα-man and replot of the slopes showing competitive mode. K_i = 16 ± 2 µM.

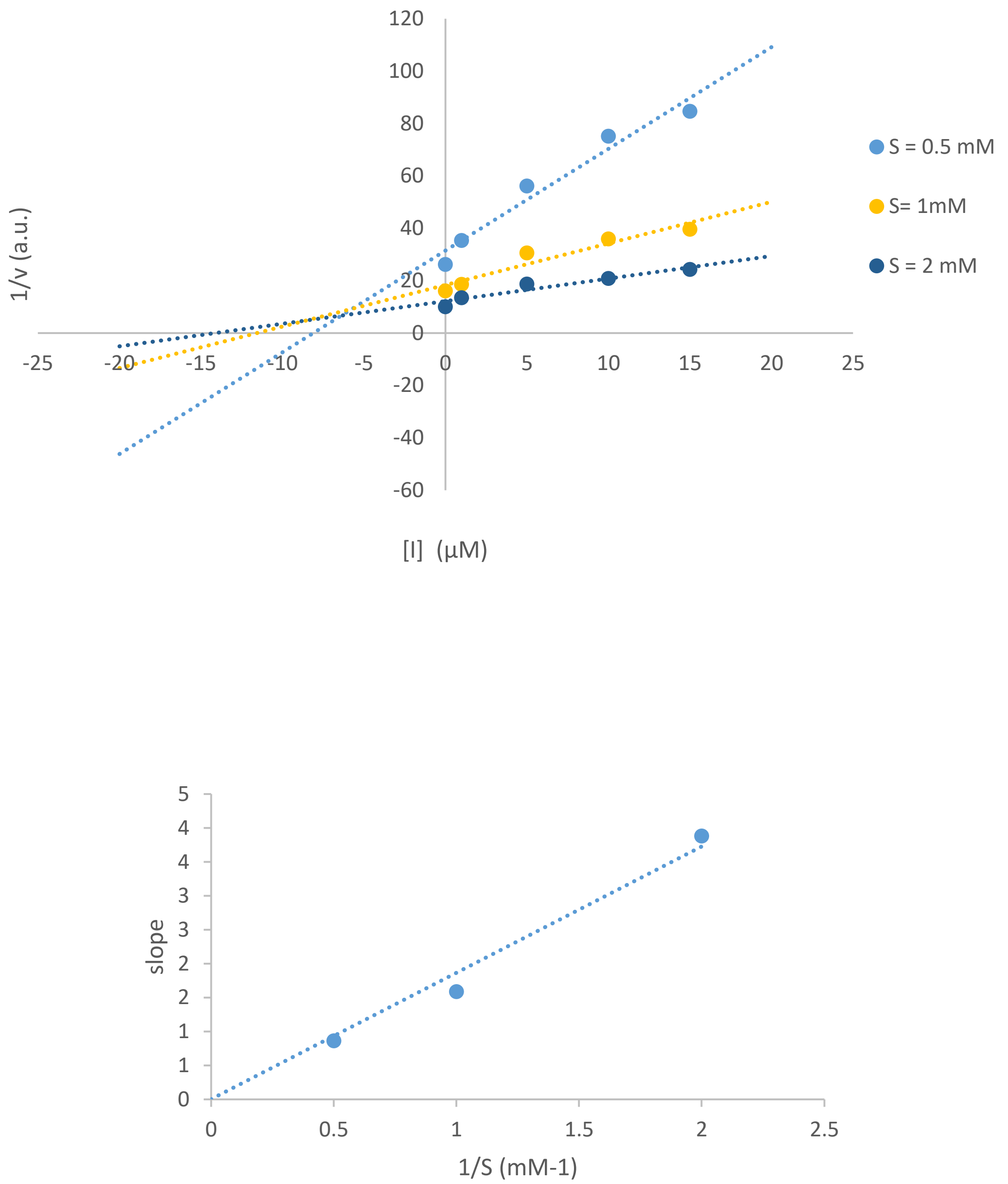

Figure S34. Dixon plot for K_i determination of compound AuGNP **2** against JBα-man and replot of the slopes showing competitive mode. K_i = 8 ± 2 μM.

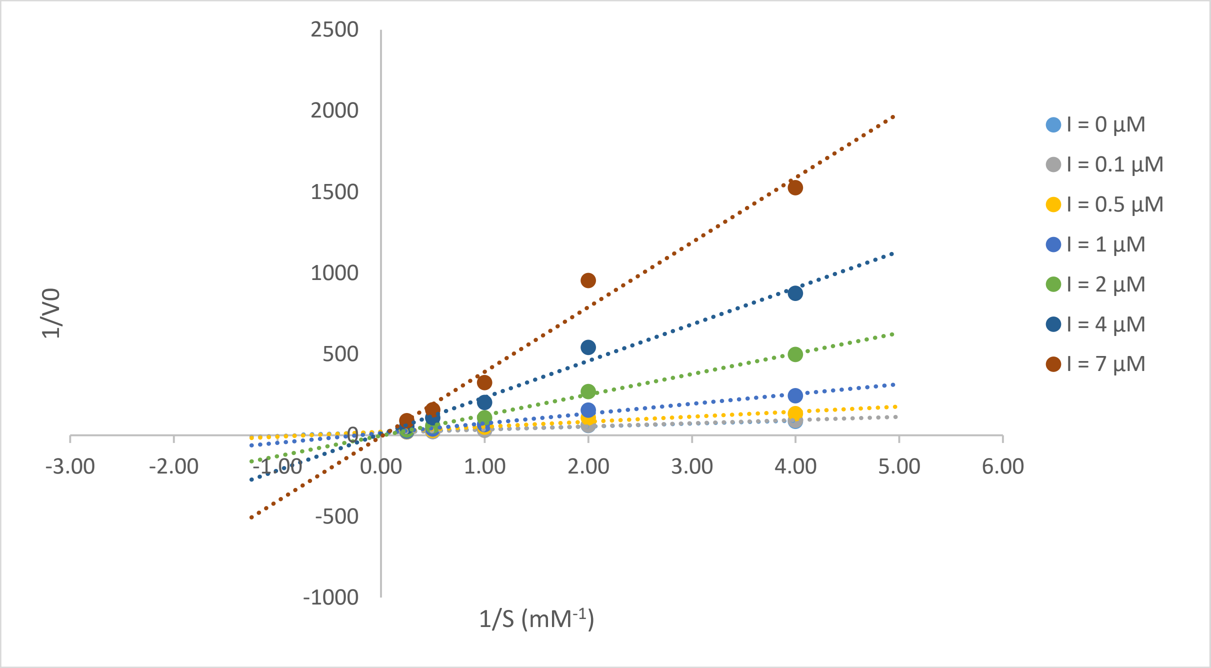

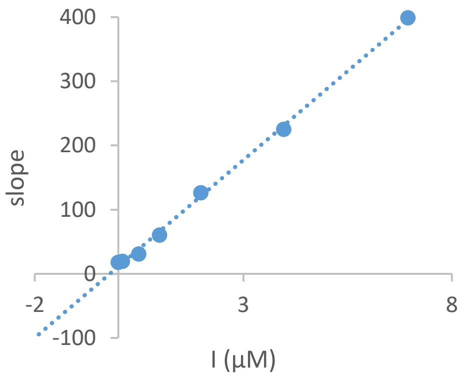

Figure S35. Lineweaver-Burk plots and replot of the slope versus inhibitor concentration for K_i determination of compound AuGNP **3** against JBα-man.
K_i = 0.198 ± 0.060 µM.

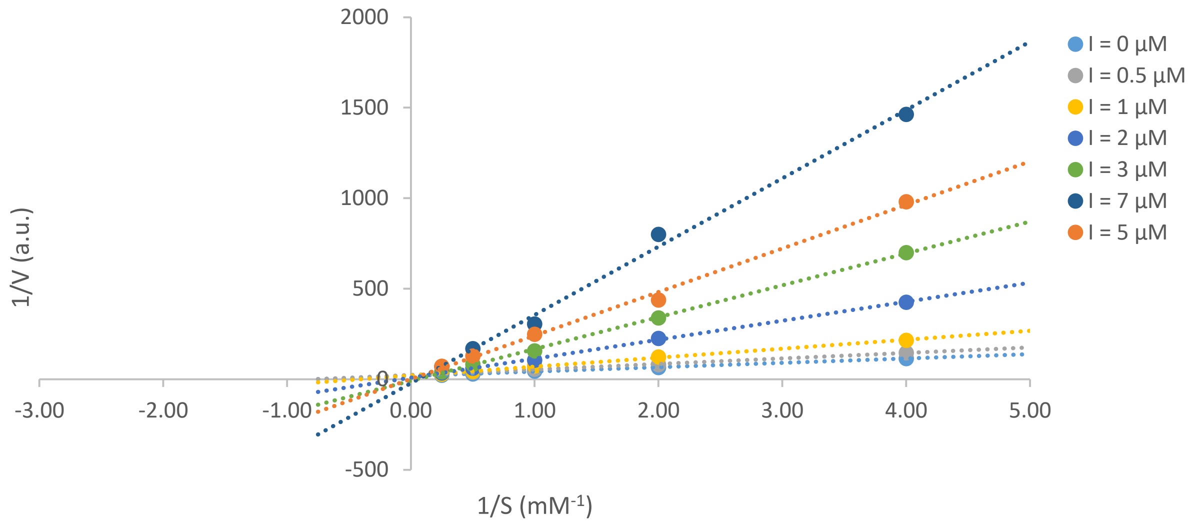

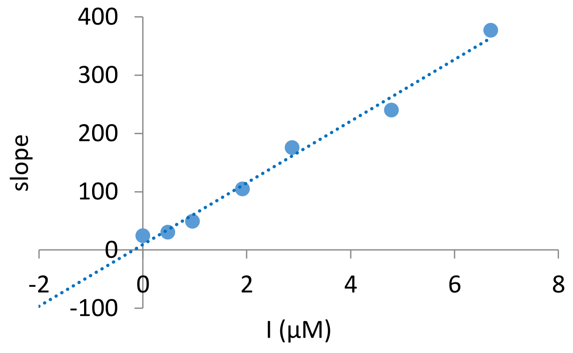

Figure S36. Lineweaver-Burk plots and replot of the slope versus inhibitor concentration for K_i determination of compound AuGNP **4** against JBα-man.
K_i = 0.175 ± 0.171 µM.

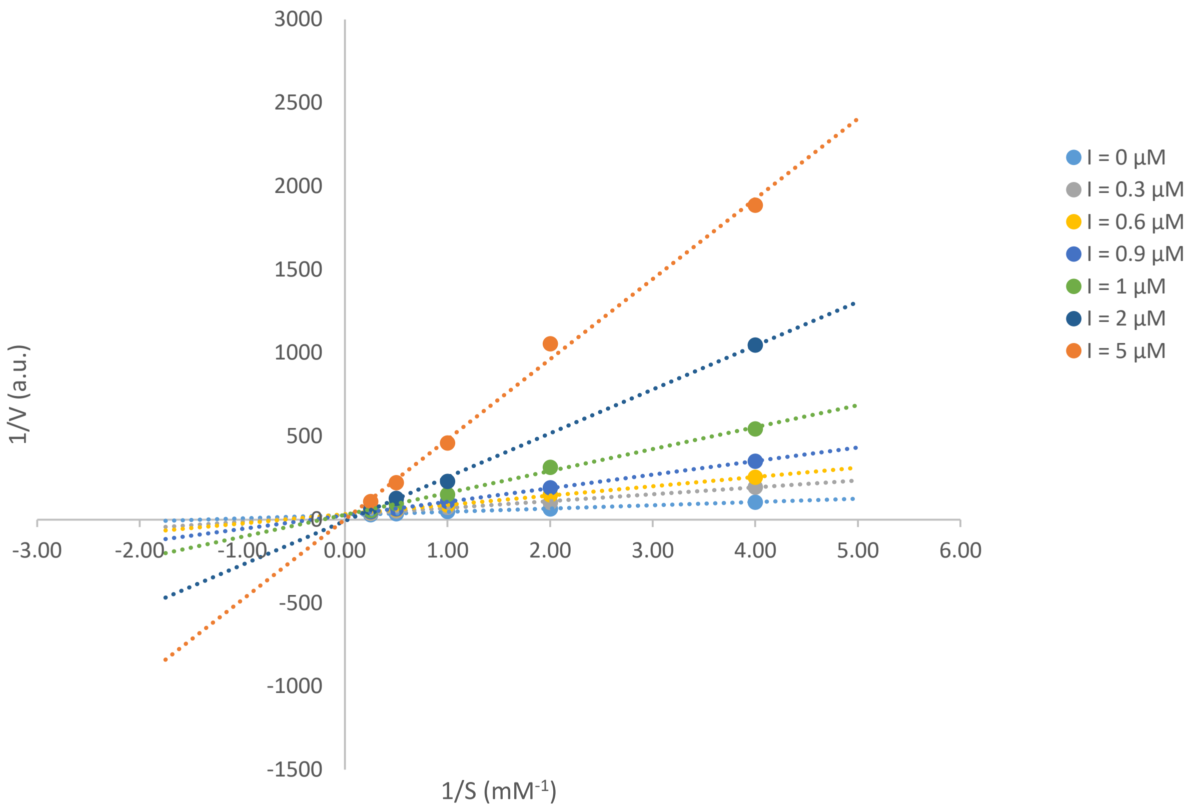

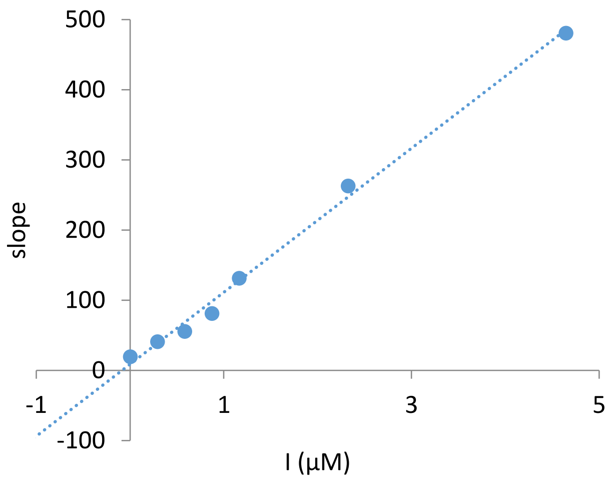

Figure S37. Lineweaver-Burk plots and replot of the slope versus inhibitor concentration for K_i determination of compound AuGNP **5** against JBα-man.
K_i = 0.084 ± 0.066 µM.
